# Genetic Diversity and Population Structure of *Fusarium commune* Causing Strawberry Root Rot in Southcentral China

**DOI:** 10.3390/genes13050899

**Published:** 2022-05-18

**Authors:** Yunlu He, Jia Chen, Chao Tang, Qiao Deng, Litao Guo, Yi Cheng, Zhimin Li, Tuhong Wang, Jianping Xu, Chunsheng Gao

**Affiliations:** 1 Institute of Bast Fiber Crops and Center of Southern Economic Crops, Chinese Academy of Agricultural Sciences, Changsha 410205, China; hyl1293416185@163.com (Y.H.); chenjia01@caas.cn (J.C.); tangchao@caas.cn (C.T.); dengqiao1234@163.com (Q.D.); guolitao@caas.cn (L.G.); chengyi@caas.cn (Y.C.); lizhimin@caas.cn (Z.L.); wangtuhong@caas.cn (T.W.); 2 Department of Biology, McMaster University, Hamilton, ON L8S 4K1, Canada

**Keywords:** fungal pathogen, root rot, simple sequence repeats, STRUCTURE, clonal dispersal, geographic structuring, genetic population, hybridization

## Abstract

Strawberry plants and fruits are vulnerable to infections by a broad range of pathogens and pests. However, knowledge about the epidemiology of pathogens causing strawberry diseases is limited. In this study, we analyzed *Fusarium commune*, a major fungal pathogen causing strawberry root rot, from diseased strawberry root tissues in southcentral China. A total of 354 isolates were obtained from 11 locations that spanned about 700 km from both south to north and east to west. Multilocus genotypes of all isolates were obtained using seven polymorphic simple sequence repeat markers developed in this study. Our analyses revealed significant genetic diversity within each of the 11 local populations of *F. commune*. STRUCTURE analysis revealed that the optimal number of genetic populations for the 354 strains was two, with most local geographic populations containing isolates in both genetic clusters. Interestingly, many isolates showed allelic ancestry to both genetic clusters, consistent with recent hybridization between the two genetic clusters. In addition, though alleles and genotypes were frequently shared among local populations, statistically significant genetic differentiations were found among the local populations. However, the observed *F. commune* population genetic distances were not correlated with geographic distances. Together, our analyses suggest that populations of *F. commune* causing strawberry root rot are likely endemic to southcentral China, with each local population containing shared and unique genetic elements. Though the observed gene flow among geographic regions was relatively low, human activities will likely accelerate pathogen dispersals, resulting in the generation of new genotypes through mating and recombination.

## 1. Introduction

The commercially cultivated strawberry (*Fragaria × ananassa*) is an economically important berry fruit crop worldwide. It is rich in vitamins, minerals, anthocyanins, antioxidants, and other nutrients, and is a highly recommended healthy food for humans [1]. Though wild strawberries are found on all continents, most are of limited agricultural and economic importance. The first strawberry plants representing the current commercial strawberry cultivars were bred in France in the mid-1700s using germplasm originated in the Americas [2]. These garden varieties were then spread across Europe and introduced to other places, including to China at the beginning of the 20th century. The area of planting strawberry has increased greatly in recent years. According to data released by the Food and Agriculture Organization (FAO), from 2008 to 2018, global strawberry production increased by 39.4%, with total production reaching about 33,276 million tons in 2019 [3]. Over the last three years, the strawberry planting area in China has been estimated at ~111,100 hm^2^, accounting for about 30% of the world’s total strawberry planting area and about 40% of the global strawberry production [4,5]. One of the fastest growing regions for strawberry production in China is in southcentral China. In addition, aside from strawberry fruit production, growing strawberry seedlings in this region for transplantation in northern China also represents an important business [6]. In southcentral China where strawberry is primarily growing in outdoor fields, the strawberry fruit ripening period is from early February (the Spring Festival season in China) to early May (the Qingming Festival). Strawberries produced during this period fill the fruit-supply gap among other major fruits and enrich the types of winter fruits for consumers. In addition, strawberries can also be produced in indoor facilities, including high-tech greenhouses, which have led to its incorporation into urban leisure agriculture, increasing its economic benefits [3,4,5,6].

Root rot is a common soil-borne disease affecting many crops and vegetables, including tomato [7], pea [8], soybean [9], yam [10], and strawberry [11]. Strawberry root rot has been reported to cause severe crop losses [12]. This disease was first reported in the UK in 1934 [13] but has ever since been reported in all countries with commercial strawberry productions [14]. In southcentral China, though strawberry root rot has been causing significant economic losses, little is known about the pathogen(s) responsible for the disease [15]. Understanding the pathogen(s) responsible for strawberry root rot can help the strawberry cultivation industry to minimize its impact, including breeding disease-resistant cultivars and developing appropriate cultivation practices and sanitation measures to control the disease [1,2,16].

The typical symptoms of diseased strawberry plants with root rot include withering, blackened fibrous roots, brown rot spots on root stems, and red or blocked main root pith [17]. The pathogens causing strawberry root rot are diverse, including species in genera *Fusarium* [18], *Dactylonectria* [19], and *Phytophthora* and *Pythium* [20]. The causal organisms can vary among strawberry production regions. For example, species *Neofusicoccum kwambonambiense* [21] and *Neopestalotiopsis rosae* [22] have only been reported in China, while in Kyrgyzstan, *Dactylonectria torresensis* was reported as strawberry root rot pathogen [23]. A major group of pathogens causing root rot is species of *Fusarium*. As the name of the disease “root rot” suggests, this disease mainly affects plant roots, but due to its impact on vascular bundle, the disease can influence the whole plant through the entire growth cycle from seedling to flowering and harvest. In severe cases, the whole plant dies, resulting in no strawberry production [16].

To prevent and control strawberry root rot, a clear understanding of its causal agents and epidemiology is needed. Since the 1990s, molecular markers have revolutionized our understanding of the epidemiology of a variety of plant, animal, and human fungal pathogens, including for identifying the origins and spread of disease outbreaks [24]. A diversity of molecular markers has been developed and used. Among them, owing to their fast mutation rate and relative ease of obtaining allelic information from a large number of strains using automated capillary genetic analyzers, simple sequence repeat (SSR) markers have emerged as the markers of choice for strain typing and for epidemiological and population genetic studies [25,26,27]. The high mutation rate makes SSR markers especially informative for analyzing recently evolved and expanding populations [28].

In this study, we aimed to understand the epidemiology of *Fusarium* root rot in strawberries in southcentral China. To achieve this objective, we collected diseased strawberry plants from 11 geographic locations in southcentral China and isolated *Fusarium* strains from diseased root tissues. Based on DNA sequences at the *Fusarium* DNA barcode locus, translation elongation factor 1-α (*tef1α*), we confirmed and selected *Fusarium commune* for further genotyping analyses. Here, we developed seven polymorphic SSR markers for *F. commune* and used these markers to genotype 354 *F. commune* strains collected for this study. The genotype information was used to identify the genetic diversity and relationships among strains and populations of *F. commune* causing strawberry root rot in southcentral China. Furthermore, this information was used to infer the epidemiology of *Fusarium* root rot and to suggest potential future areas of research.

## 2. Materials and Methods

### 2.1. Sample Collection

**Figure 1:**
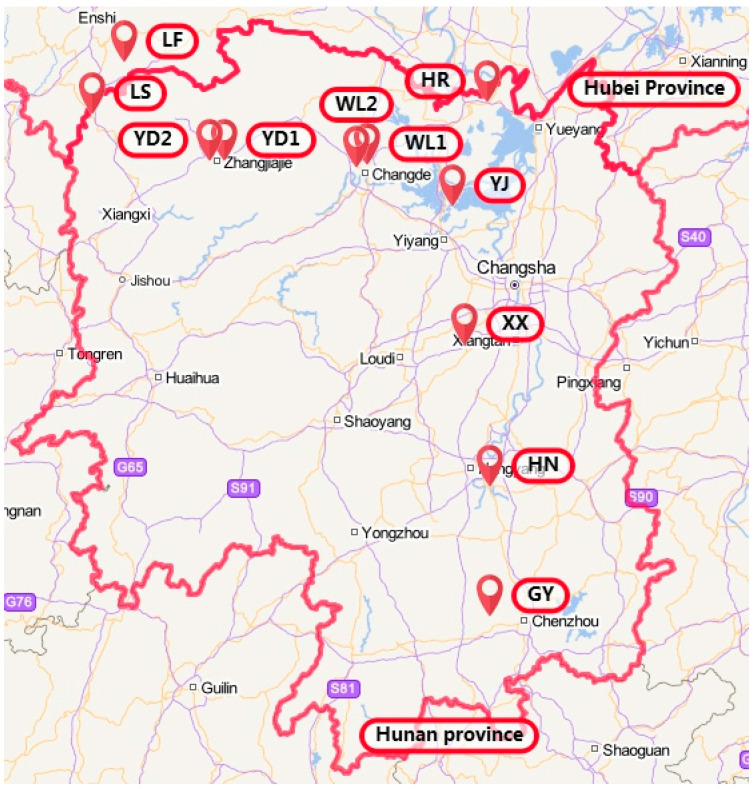
Geographical distribution of 11 strawberry root rot sampling. This figure was generated using the Landmap program (https://www.ldmap.net/index.html, accessed on 15 March 2022) with input of the geographic coordinates for the 11 sites.

From April to May 2019, we collected strawberry plants with typical symptoms of root rot disease (wilting and discolored roots) from 11 sites in the following nine counties/municipalities in southcentral China: Chenzhou, Hengyang, Xiangtan, Yueyang, Yiyang, Changde, Zhangjiajie, and Xiangxi in Hunan Province, and Laifeng in Hubei Province. The sampling sites spanned from Chenzhou, Hunan, in the south to Yueyang in the north, and from easternmost Hengyang and Chenzhou to Xiangxi in Hunan and Hubei’s Laifeng in the west. The sampled sites included a variety of terrains and climates (Figure 1). Details about these sites are presented in Table 1, including their location, latitude and longitude, time of sampling, and abbreviated code used elsewhere in this study. Among these 11 sites, three (GY, HN, and XX) are situated in the lowland rolling hill regions; four (YJ, HR, WL1, and WL2) are in the flood plains of Dongting Lake; and four (YD1, YD2, LS, and LF) are in the mountains. At each site, ten wilted plants were collected, with individual diseased plants located at least 5 m from each other. The diseased plants were taken back to the laboratory for cleaning and screened for pathological features associated with root rot, including blackened fibrous roots, brown rot spots on root stems, and red or blocked main root pith. The confirmed diseased plants were stored in the refrigerator at 4 °C until the tissue samples were processed for isolating the putative infecting pathogens.

### 2.2. Isolation of F. commune Strains

To isolate the infecting pathogen, the strawberry plant root system was divided into three parts, corresponding to fibrous roots, main root epidermis, and main root stem pith. From each of the 11 sites, 30 pieces of diseased tissue were taken for each of the three root tissue types for pathogen isolation. Here, for samples from fibrous roots, each piece of diseased tissue represented a distinct infected fibrous root. For samples from main root epidermis, each piece of diseased tissue represented a distinct site along the main root. Similarly, for samples from the main root pith, each piece of diseased tissue represented a distinct section within the main root stem. Up to five pieces of infected tissues from each plant for each of the three root tissue types were used for isolating the infecting pathogen. The different root tissue samples were used to investigate whether there were tissue-specific differences among the alleles and genotypes of the infecting pathogen populations.

For pathogen isolation, strawberry roots with typical symptoms of root rot were washed with clean tap water to remove soil remnants and soft rotten tissues. Each diseased tissue was then surface sterilized with 75% ethanol for 1 min, followed by soaking in 2% sodium hypochlorite solution for 10 min, and washed with sterile pure water 3 times. The surface-sterilized diseased tissue was then dried using sterile filter paper, cut using a sterile scalpel into small pieces of about 0.01 cm^3^, transferred onto a potato dextrose agar (PDA) medium plate containing 50 μg/mL streptomycin, and placed in an incubator at 25 °C. Three tissue pieces were placed on each plate. When the fungal colony grew to about 2 cm in diameter, a sterile toothpick was used to pick a small clump of hyphae from the edge to cultivate pure culture. Fungal colonies with vigorously growing aerial hyphae and macroconidia resembling a spindle were selected for downstream analyses.

### 2.3. DNA Extraction, PCR Amplification, and Species Identification

For species identification, we followed the method of O’Donnell et al. [29] and obtained the DNA sequence of the translation elongation factor 1 α subunit (*tef1α*) gene from each isolate. Briefly, mycelia were collected from 5-day-old cultures grown on PDA for each *Fusarium* isolate. The total genomic DNA was extracted following the protocol described in Fang et al. [10]. PCR amplification was performed in a final volume of 25 µL containing 1 µL genomic DNA, 1 µL upstream and downstream primers (10 nM), and 12.5 µL Taq PCR MasterMix (Shengong Bioengineering Co., Ltd., Shanghai, China). The *tef1α* barcode region was amplified with primers EF1 (5′ ATGGTAAGGARGAGAAC 3′) and EF2 (5′ GGARGTCAGTSATCATG 3′). PCR amplification was performed in a T100TM PCR instrument (Bio-Rad Laboratories, Hercules, Wilmington, DE, USA). The initial denaturation was performed at 94 °C for 5 min, followed by 35 amplification cycles. Each amplification cycle included denaturation at 95 °C for 30 s, annealing at 55 °C for 30 s, extension at 72 °C for 1 min, and finally extension at 72 °C for 10 min. The amplification was verified by agarose gel electrophoresis (1× agarose buffer). The amplified PCR products were purified and sequenced by Tsingke Biotechnology (Changsha, China).

The *tef1α* DNA sequence obtained from each isolate was compared with those in the GenBank database. After BLAST comparison, we retrieved the representative GenBank *tef1α* sequences of *Fusarium* species that were closely related to our sequences, especially those within the *Fusarium oxysporum* species complex. These retrieved sequences were combined with our own sequences for phylogenetic analysis to confirm the species identity of our strains, using the MEGA 7.0 software [30]. The maximum likelihood (ML) algorithm was used to generate the phylogenetic tree. Branch support was inferred from 1000 bootstrap repetitions, and gaps in sequence alignment due to nucleotide insertions and deletion were excluded from analysis [30]. The output phylogenetic tree was edited using the online drawing tool iTOL (Interactive Tree of Life, https://itol.embl.de, accessed on 15 March 2022) [31].

### 2.4. Development of SSR Markers for F. commune

Because there is currently no reported multilocus polymorphic marker for *F. commune*, we first developed new SSR markers for genotyping *F. commune* strains. To develop polymorphic SSR markers, we first retrieved the genome sequence of *F. commune* from NCBI. The *F. commune* genome data were mined using the MISA v2.1 (http://pgrc.ipk-gatersleben.de/misa/misa.html, accessed on 1 April 2021) program. The default setting of the MISA program defined SSR markers as containing one of the following: 1 base repetitions ≥10 times; 2 base repetitions ≥6 times; 3 base, 4 base, 5 base, and 6 base repetitions ≥5 times. When the distance between two SSR markers is less than 100 bp, they form a composite SSR marker and were included as a single SSR marker for testing. After SSR marker identification, we used Primer 3.0 (http://primer3.sourceforge.net/) software (version 2.5.0, accessed on 15 April 2021) to design primers (default parameters).

To identify potential polymorphic markers for our strains of *F. commune*, we selected eight strains representing different *tef1α* sequence types and screened 30 pairs of SSR primers, following protocols described previously [32]. The information about the 30 primer pairs, including their amplification success and allele numbers among the eight strains, is summarized in Supplementary Table S1. All primers were synthesized by Tsingke Biotechnology (Changsha, China). The primer pairs showing 100% amplification success and with >4 alleles each were chosen for further genotyping the total population. The following seven primer pairs met the criteria: CM15, CM18, CM22, CM23, CM25, CM27, and CM28. The primer sequences and other information about these seven SSR markers are presented in Table 2.

### 2.5. SSR Genotyping for F. commune Isolates

To obtain multilocus genotype information for all strains in the population, one primer in each of the seven SSR primer pairs was fluorescently labeled with FAM or HEX (Tsingke Biotechnology). For each isolate at each SSR marker, the PCR reaction (15 µL) consisted of 7.5 µL 2× Tsingke MasterMix, 1 µL of each primer (10 nM), and 1 µL template DNA. PCR cycles consisted of a 5 min denaturation (at 94 ℃), followed by 30 cycles of denaturation (94 ℃), annealing (50–55 ℃), and extension (at 72 ℃), each for 30 s. PCRs were terminated after a final 5 min extension at 72 ℃. Successful amplifications of the SSR markers were confirmed by agarose gel electrophoresis. After confirmation, 5 µL of each amplification product was mixed with 10 µL of a mixture containing highly deionized formamide and rox-500 fluorescent molecular-weight internal standard as recommended by the manufacturer. The mixtures were then denatured at 95 ℃ for 5 min, placed on ice for 10 min, and loaded onto an ABI 3730xl automatic DNA analyzer for capillary electrophoresis. The output files were analyzed using GeneMapper 4.1 software to identify the lengths of amplified fragments of *F. commune* isolates and using fluorescent marker standards for each run as fragment size references and following the default setting as recommended by the program. An example graph for allele calling is shown in Supplementary Figure S1.

### 2.6. Data Analysis

The strain relationships based on SSR genotype data were inferred based on the UPGMA algorithm using MEGA7 software. For each of the 11 local populations, the mean numbers per locus for the following diversity features were calculated using PowerMarker v3.25: (i) observed alleles, (ii) alleles with a frequency ≥5%, (iii) effective alleles, (iv) the Shannon’s diversity index, (v) private alleles, (vi) locally common alleles (Freq. ≥5%) found in 25% or fewer populations, (vii) locally common alleles (Freq. ≥5%) found in 50% or fewer populations, (viii) expected heterozygosity, and (ix) unbiased expected heterozygosity. The optimal number of genetic populations (K) in the total sample was inferred by STRUCTURE 2.3.4. The STRUCTURE analysis used the admixture model, with 10 replicated runs of K = 1 to 10 after an initial burn-in of 100,000 generations followed by a run length of 1,000,000 generations. The optimal K value was identified following the method described in Evanno et al. [33], using Structure Harvester (http://taylor0.biology.ucla.edu/structureHarvester, accessed on 10 January 2022).

For genetic analyses between local geographic populations such as the analysis of molecular variance (AMOVA), pairwise population *F_ST_*, and principal components, we used GenAlEx v 6.5 software [34]. Two types of samples were investigated in our population genetic analyses. The first sample type included all 354 strains. The second sample type used clone-corrected sample where only one representative strain of each multilocus SSR genotype was included in each local geographic population. The three types of root-tissue-based *F. commune* samples (fibrous root, main root epidermis, main rhizome pith) were similarly analyzed to identify their potential contributions to the total observed genetic variation. The overall relationship between geographic and genetic distances between all pairs of the 354 strains was analyzed using the nonparametric Mantel test through GenAlEx v6.5 software (Melbourne, Australia) [34].

## 3. Results

### 3.1. Isolation and Molecular Identification of F. commune by tef1α Sequencing

**Figure 2:**
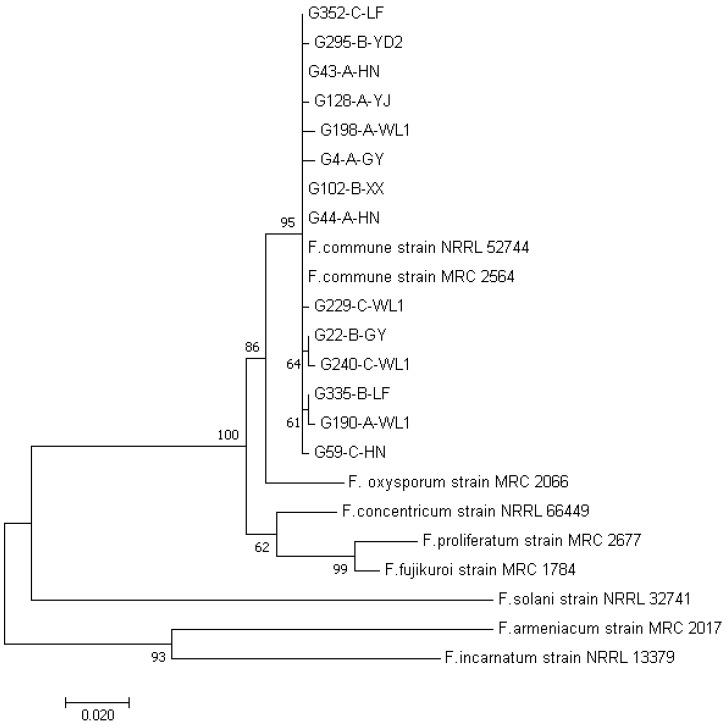
Phylogenetic tree showing the relationships between representatives of our *F. commune* strains and those of closely related species based on *tef1α* sequences. Only one representative strain for each of the 14 *tef1α* sequence types observed in our total sample was included in this figure.

Based on colony and microscopic morphological characteristics and DNA sequences at the *tef1α* locus, we obtained a total of 354 *F. commune* isolates. The number of *F. commune* isolates from each location is shown in Table 3. Among the 11 geographic locations, the number of isolates ranged from 11 in Longshan (LS) to 66 in Langzhou, Wuling District (WL1). *F. commune* strains were isolated from all three types of root tissues, with 174 strains from full fibrous roots, 120 from the main root epidermis, and 60 from the main rhizome pith. The *tef1α* sequence of all 354 *F. commune* isolates have been deposited in GenBank under accession numbers MZ041792–MZ042021. Figure 2 shows the relationships between our representative strains for the 14 *tef1α* sequence types and those of closely related *Fusarium* species based on *tef1α* sequences. The relationships among all 354 strains based on *tef1α* sequences are shown in Supplementary Figure S2.

### 3.2. SSR Marker Development

Using MISA to screen the whole-genome sequence of *F. commune*, we identified a total of 2432 SSR loci belonging to the nine types of SSR. The most abundant type was single nucleotide repeats (46.6%), followed by di- (19.2%), tri- (20.4%), quad- (4.2%), penta- (3.0%), and hexa- (1.8%) nucleotide repeats. From these, we randomly chose 30 to design primer pairs to screen for potential polymorphisms using eight geographically diverse strains from our collection. Among these 30 SSR markers, seven showed 100% amplification success, an unambiguous single amplified product for each strain at each marker, and more than four alleles each among the eight tested strains (Supplementary Table S1). Information about the seven SSR loci, including their primer sequences, amplified fragment lengths, repeat types, and allelic lengths are shown in Table 2. BLAST analyses of the seven SSR loci sequences against the assembled *F. commune* genome sequence showed that these seven SSR loci were all located on different contigs. Among these seven loci, their amplified fragment sizes ranged from ~100 to ~400 bp. These seven pairs of primers were then used to analyze our samples of 354 *F. commune* strains.

### 3.3. Allele and Genotype Characteristics of the Seven SSR Markers

Our analyses revealed that a single PCR-amplified product was detected at each SSR locus in each of the 354 isolates, consistent with all strains being haploid and that each amplified sequence was likely present in a single copy in each isolate. In the total sample of 354 isolates, the average number of alleles was 16 per locus, ranging from six (for marker locus CM23) to 36 (for marker locus CM22) (Table 2). Together, the seven SSR marker loci revealed a total of 115 alleles across the seven loci. All seven SSR loci were polymorphic in all 11 geographic populations.

The seven SSR markers identified 158 multilocus genotypes (MLGs) among the 354 isolates (Supplementary Table S2 and Figure S3). Among the 158 MLGs, 94 were represented by one isolate each while the remaining 64 MLGs were each represented by two or more isolates in our sample. Among these 64 MLGs, 52 were each represented by two or more isolates from the same geographic location only and together these 52 MLGs represented 188 isolates total in our sample. The most frequent MLG was MLG#20, shared by 19 isolates with all of them from Xiangxiang County. For the remaining 12 MLGs, 10 were shared between two of the 11 local populations and included a total of 54 isolates. The remaining two MLGs were each shared among three geographic populations and together they represented a total of 18 isolates. The longest-distance (~700 km) shared genotypes were between Laifeng County in Hubei and Guiyang County in Hunan, where three MLGs were shared (MLG#33, #96, and #103) with MLG#103 shared among 17 isolates between these two locations. The relationships among all 354 isolates based on the SSR genotypes, including the distributions of the shared MLGs among geographic and root tissue samples, are depicted in Supplementary Figure S3 and Table S2.

### 3.4. Allelic and Genotypic Variations within Geographical Populations of F. commune

Our analyses revealed that overall, there was considerable genetic diversity of *F. commune* causing strawberry root rot within individual local populations as well as in the total sample in southcentral China. For each of the seven SSR loci, two or more alleles were found within each of the 11 local geographic populations. In addition, private allele(s) were found within 10 of the 11 local geographic populations. The only exception was YD2, where no private allele was found while in the WL1 population a total of nine private alleles were found at the seven SSR loci. Overall, among the 11 local populations, the YD1 population had the lowest numbers of observed alleles and effective alleles, and the lowest gene diversity per locus. In contrast, the GY population had the highest number of observed alleles (7.9 per marker locus) while the YJ population had the highest number of effective alleles (4.3 per marker locus) and the highest expected heterozygosity within the population. The details about allelic richness and allelic frequency distribution patterns within each of the 11 local geographic populations are shown in Table 4. Together, our analyses revealed that there was high-level allelic diversity, with indigenous genetic elements within most of the 11 local populations of *F. commune* analyzed here.

Similar to the diversity of alleles observed within each geographic population, a range of MLGs was found among the 11 local populations of *F. commune*. The lowest number of MLGs was found in the LS and YD1 populations (with 7 MLGs each) while the highest was found in the WL1 population (28 MLGs). However, the observed variation in the number of MLGs was likely due to sample size differences among the local populations because the number of MLGs was positively correlated with sample size (Pearson correlation coefficient = 0.88, *p* < 0.05). As described in the previous section, among the total 158 MLGs, most (i.e., 146 of the 158 MLGs) were only found in one of the 11 local populations while 12 were shared between two to three local populations. Two local populations (XX and YD1) shared no MLG with other geographic populations. These two local populations were about 300 km from each other and located close to the middle of our sampled regions (Figure 1). In contrast, the LF population located at the northwest corner of our sampled region contained eight shared MLGs with other local populations, including the GY population located in the southeast corner of our sampled sites. The other eight local populations contained 1–4 shared MLGs each. The total number of MLGs, private number of MLGs, and shared number of MLGs within each of the local population are shown in Supplementary Table S3.

### 3.5. Relationships among Geographic Populations of F. commune

In this study, the relationships among populations based on allele and genotype frequencies were analyzed for two sample types, one included the total samples and the second included clone-corrected samples. In the total sample without clone-correction, the analysis of molecular variance (AMOVA) revealed that geographic separation among the 11 local populations contributed 13% to the total genetic variance while individual populations contributed 87%. Both the within- and among-population contributions to the total genetic variation were statistically significant (*p* < 0.001). Though still statistically significant (*p* < 0.001), after clone-correction the percentage of contribution to the total genetic variance by geographic separation was reduced to 6% (Table 5).

In the pairwise local population comparisons without clone-correction, we found that all 55 ((11 × 10)/2 = 55) pairwise comparisons showed statistically significant differences at *p* < 0.05, with 53 of the 55 total pairwise population comparison *p* values smaller than 0.001 (Table 6). The pairwise *F_ST_* values ranged from 0.036 (between LS and WL2) to 0.333 (between YD1 and XX). Here, the *F_ST_* values of 0.036 and 0.333 represent the percentages (3.6% and 33.3% respectively) of the total genetic variance contributed by geographic separation between pairs of local population. Overall, based on the total sample of 354 isolates, the YD1 population showed the highest genetic differentiation from other geographic populations (Table 6). The clone-corrected sample showed a similar pattern with all pairwise comparisons showing statistically significant genetic differentiations from each other and with the YD1 population being the overall most differentiated from other geographic populations (Table 7). However, compared to the total samples without clone-correction, all the pairwise *F_ST_* values in the clone-corrected sample were lower.

**Figure 3:**
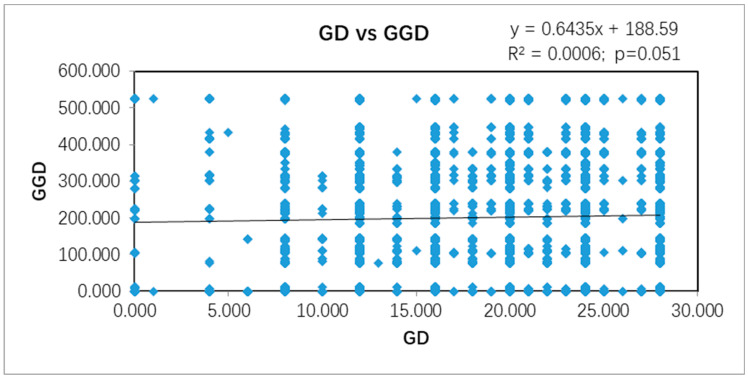
Mantel test between geographic and genetic distances between all pairs of strains analyzed in this study. A total of 354 isolates were included in this analysis. GD: genetic distance; GGD: geographic distance (km).

**Figure 4:**
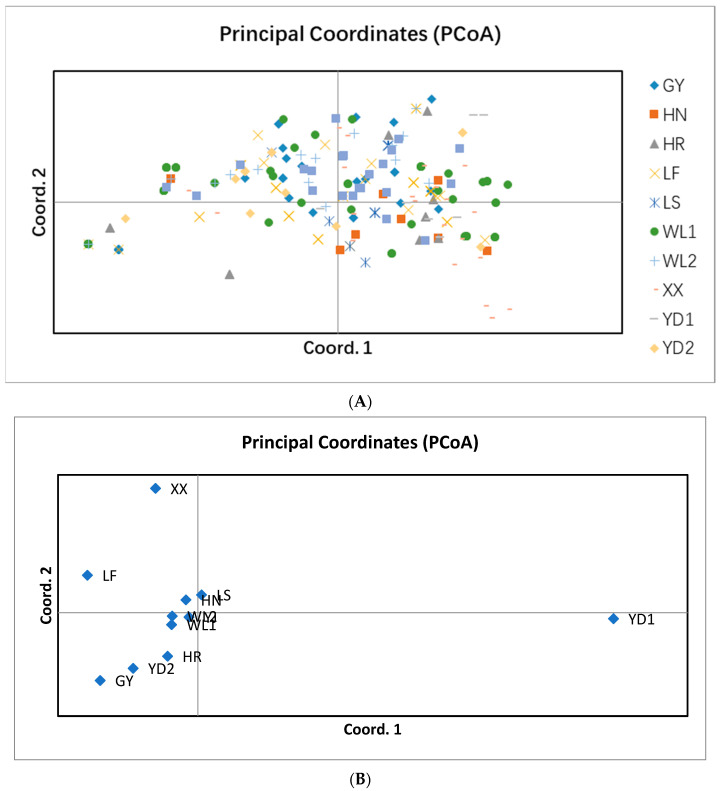
Principal coordinate analysis (PCoA) of the genetic variation among strains and populations of *F. commune* analyzed in this study. (**A**): PCoA based on 354 strains; (**B**): PCoA based on 11 local geographic populations.

While statistically significant genetic differentiation was found among geographic populations, there was no evidence for significant correlation between genetic difference between strains of *F. commune* in southcentral China and their geographic distances from each other. Specifically, in the strain-based Mantel test, the *R*^2^ value was 0.0006 and the *p* value was 0.051 (Figure 3). A principal coordinate analysis also showed an overall mixing of strains from most geographic areas (Figure 4A). However, small clusters of strains from individual geographic populations were found in the PCoA graph (Figure 4A), a result consistent with the genotype-sharing among strains being predominantly from within individual geographic locations as described earlier (Supplementary Figure S3 and Table S2). When PCoA analyses were conducted at the local geographic population level, the separation of geographic populations from each other was clearer than that based on individual strains (Figure 4B). In Figure 4B, local population YD1 was distinctly separated from other 10 location populations in the first coordinate.

Different from the significant geographic contributions to the genetic variation, we found no statistically significant evidence for root tissue-based genetic differences in our samples of *F. commune* in southcentral China. Specifically, in both the total and the clone-corrected samples, less than 1% of the total genetic variance could be attributed to the separation of the samples into three root tissue types (Table 5, *p* > 0.10). Together, this result suggests no evidence of strawberry root tissue-specific specialization for strains of *F. commune* in southcentral China.

### 3.6. STRUCTURE Results

**Figure 5:**
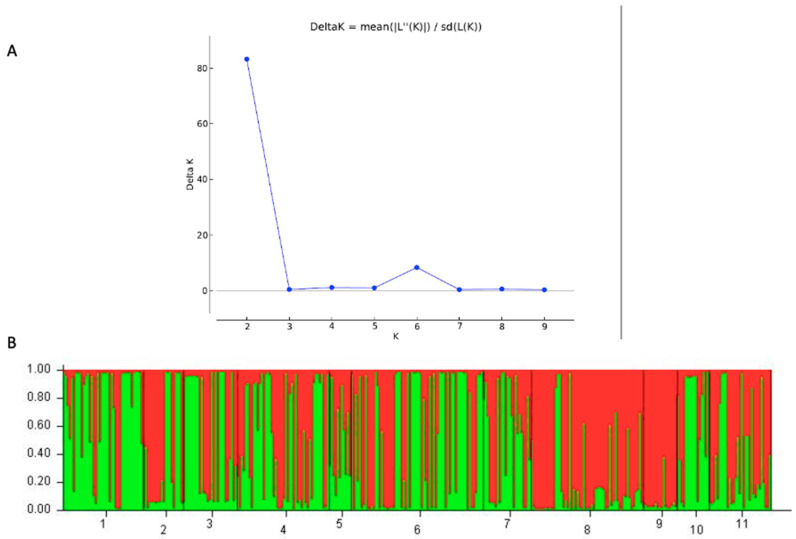
Results from STRUCTURE analyses of 354 isolates of *F. commune* causing root rot in strawberries in southcentral China. (**A**) shows evidence for optimal K value of 2. (**B**) shows the distribution of two genetic clusters among 11 local populations. The population labels of 1–11 on the X-axis correspond to GY, HN, HR, LF, LS, WL1, WL2, XX, YD1, YD2, and YJ, respectively.

Bayesian clustering using STRUCTURE revealed that the estimated optimal K value was two for the 354 *F. commune* isolates (Figure 5). The analysis showed that within each geographic population, there were genetic elements of both genotypic clusters. However, the distributions of isolates belonging to these two genetic clusters differed among the geographic populations. For example, isolates of the YD1 and XX geographic populations predominantly belonged to one genetic cluster (shown as red in Figure 5) while those in the GY and YD2 geographic populations mainly belonged to a different genetic cluster (shown as green). Interestingly, many isolates contained alleles of both genetic clusters. We note that the second-best estimated optimal K value was six (Figure 5A). Similar to that observed for when K = 2, most local geographic populations contained strains belonging to different genetic clusters, including many strains with mixed ancestries (i.e., those with a mixture of colors; Figure 5B and Figure S4).

## 4. Discussion

This study developed an SSR genotyping system for *F. commune* and investigated the diversity and distributions of SSR alleles and multilocus genotypes of *F. commune* samples causing strawberry root rots in southcentral China. Our analyses revealed abundant genetic variation within each of the 11 local geographic populations. Though multilocus genotype sharing was found between strains from several different local populations, all 11 local populations were statistically significantly differentiated from each other. However, the levels of genetic differentiation were not significantly correlated with geographic distance. STRUCTURE analysis revealed that the total sample consisted of two genetically distinct clusters, and with all 11 local populations containing elements of both genetic clusters, including many isolates showing evidence of having genetic ancestries from both clusters. Together, our results suggest that the populations of *F. commune* causing strawberry root rot are highly genetically diverse within most locations in southcentral China. However, gene flow has occurred among the local populations.

Before the name *F. commune* was introduced as a separate new species in 2003, strains of *F. commune* were identified and reported as *F. oxysporum* or the *F. oxysporum* species complex [35]. However, since its description in 2003, *F. commune* has been reported from many parts of the world, including the US, Japan, and China. This fungal pathogen has been found to cause diseases in a variety of agricultural crops and vegetables such as root rot in lotus, tobacco, potato, kiwi, rice, and cowpeas, leading to significant crop yield losses [36,37,38,39,40,41]. However, so far, *F. commune* has not been reported to cause strawberry root rot. In contrast, in California, *F. oxysporum* f. sp. *fragariae* has been reported to cause of *Fusarium* wilt of strawberry. It should be noted that the absence of report linking *F. commune* to strawberry root rot does not mean that *F. commune* has not caused strawberry root rot. We believe that it is highly likely that some of the previously (before 2003) identified strains of “*F. oxysporum*” causing strawberry diseases (including root rot) likely belonged to *F. commune*. Regardless, our results here clearly indicated that a diversity of *F. commune* genotypes can cause strawberry root rot.

Molecular markers such as SSR have been increasingly used for studying the origins and spread of plant fungal pathogens, including tracking the pathogens responsible for crop disease outbreaks, epidemics, and pandemics [42]. For *Fusarium* species, SSR markers have been used to study the epidemiology of several plant pathogens such as *F. proliferatum* [43], *F. culmorum* [44], *F. oxysporum*, *F. fujimorium* [10], and *F. mexicanum* [45]. SSR markers are based on copy number variations in one or a few nucleotide repeats within specific genomic regions. They typically have much higher mutation rate than base substitutions. Consequently, they have been extensively used for studying the patterns of recent migrations and dispersals [42]. In this study, SSR molecular markers were used for the first time to study the genetic diversity of *F. commune* populations. The results showed that these seven SSR markers were highly polymorphic and together, they helped reveal the diversity and distributions of *F. commune* genotypes across 11 local populations in southcentral China. We note that within the seven SSR markers, aside from the nucleotide repeat number differences among alleles, single nucleotide insertions/deletions outside of the repeated regions but within our amplified DNA fragments were also present (Table 2). Such differences were detected using an automatic DNA analyzer through capillary electrophoresis, contributing to allelic polymorphisms at all seven loci. The seven SSR markers developed here should be able to help future studies of *F. commune* impacting strawberries in other parts of the world and affecting other crops within and outside of southcentral China.

Overall, our analyses identified many local population-specific genotypes and a few shared ones between/among the 11 local populations. The shared genotypes likely represent recent gene flows between/among local populations. As described previously and above, *F. commune* can grow saprophytically in the soil and on a diversity of plants [42,46]. Consequently, gene flow in *F. commune* could be mediated by both natural factors and anthropogenic activities. For example, under certain conditions, *F. commune* can produce abundant asexual spores (conidia) that can easily become airborne and disperse by wind. However, if wind-aided natural dispersal were prevalent, we should expect a significant correlation between genetic distance and geographic distance among our strains. The absence of such a statistically significant correlation between genetic and geographic distances suggest that wind-aided dispersals were likely uncommon. Alternatively, human travel between regions, strawberry seedling trade, and other agricultural activities could bring strains of *F. commune* from one location to another. The longest-distance shared genotypes identified here between the GY and LF populations (about 700 km from each other; Figure 1) were likely mediated through anthropogenic activities. However, these two possibilities are not mutually exclusive, and both could have contributed to the observed genotype-sharing. Overall, our current data reported here indicated that anthropogenic activities likely contributed more than wind-aided dispersal to the observed genotype-sharing among *F. commune* strains causing strawberry root rot in southcentral China.

Among the 11 local geographic populations of *F. commune* from southcentral China, the YD1 populations were the most distinct from others. No strain in the YD1 population shared a multilocus genotype with those in any other geographic population (Supplementary Figure S3 and Table S2). YD1 also had the lowest mean number alleles per locus, the lowest number of effective alleles, the lowest expected heterozygosity, and the lowest number of multilocus genotypes (Table 4 and Table S2). At present, the reason(s) for the distinctiveness of the YD1 population is not known. This population was similar to several other local populations in both geographic and ecological characteristics (Figure 1 and Table 1). Broader sampling is needed in order to understand the potential contributors to the genetic distinctiveness of the YD1 population.

The observed high allelic and genotypic diversities are consistent with the local populations of *F. commune* existed long before strawberries were introduced to southcentral China less than 100 years ago. Thus, the strawberry root rot caused by these pathogens most likely represents a (relatively) recent shift from a saprophyte or a pathogen on other plants/crops to strawberries. Indeed, the two or more genetic clusters identified in our sample and the diverse genotypes found in most geographic populations suggest that multiple switches by *F. commune* have likely occurred in southcentral China to colonize and infect strawberries. Furthermore, the differential distributions of different MLGs, with some being more common and more broadly distributed than others, suggest that some of the *F. commune* genotypes identified here are likely more adapted to strawberries than other *F. commune* genotypes. Whether the frequently observed genotypes of *F. commune* are also more frequently found in the soil and/or in other plants at these sites remain to be investigated. In a study of *Fusarium graminearum* from different sources in Lithuania and Latvia, low but statistically significant genetic difference (raw data, *F_ST_* = 0.0245, *p* < 0.05; clone-corrected data, *F_ST_* = 0.0245, *p* < 0.05) were found between the soil population and those from living plants [47]. However, no statistically significant difference was found among *F. graminearum* populations from different groups of host plants or from different years. Their results suggested evidence for potential differential infectiousness/pathogenicity among strains in the soil population of *F. graminearum* [47]. At present, whether a similar phenomenon exists in *F. commune* in their pathogenicity toward strawberries is not known. To address this question, comparable number of soil strains of *F. commune* from each of the 11 corresponding strawberry fields analyzed here will need to be isolated and analyzed using the same molecular markers for comparison. In addition, if differences were found, the differentially distributed alleles and genotypes of *F. commune* strains between these two ecological niches should be tested to confirm their potential pathogenicity differences on strawberries. Regardless, the high-frequency and broadly distributed genotypes identified in our study represent ideal materials from which to investigate the genetic bases of *F. commune* pathogenicity causing strawberry root rot and as priority test genotypes for developing root rot-resistant strawberries.

Because of structural differences among the three types of root tissues in strawberries, we hypothesized that strains of *F. commune* infecting each tissue type might be genetically different from the other two types. However, based on the seven SSR markers, no evidence for significant genetic difference was found among the three types of samples of *F. commune* based on their infecting root tissue types in southcentral China. It should be noted that our data (i.e., the lack of genetic differentiation among *F. commune* samples from the three root tissue types) do not exclude the possibility that there might be genes in *F. commune* responsible for a certain degree of root tissue-specific trophism and pathogenesis. Interestingly, despite relatively equal efforts spent in trying to isolate *F. commune* from the three root tissue types, the isolation success ratio from fibrous roots, the main root epidermis, and the main rhizome pith was about 3:2:1. This ratio likely reflects the infection process of strawberry root rot where fibrous roots are likely the easiest for the pathogen to infect and grow inside, followed by the main root epidermis, and then inside the main rhizome. However, the frequent sharing of *F. commune* genotypes among the three root tissues and the lack of overall genetic differentiation among them are consistent with most *F. commune* strains and genotypes identified here being capable of infecting all three strawberry root tissues in southcentral China.

In summary, our study provides the first insights into the genetic diversity and population structure of *F. commune* causing strawberry root rot in southcentral China. Our analyses revealed high genetic diversity and significant differences among the 11 local populations of *F. commune*. The SSR markers developed here should help facilitate future studies on the epidemiology of strawberry root rot in other geographic regions and the relationships between *F. commune* populations from strawberries and other crops. Information from such studies could help identify the origins and route(s) of spread of genotypes, especially the high-frequency virulent genotypes, which in turn can help design better strategies to minimize their spread and disease impact.

## Figures and Tables

**Table 1:** Geographic information for the 11 sampling sites, sampling time, and abbreviated code for each. The sample sizes of *F. commune* strains obtained at the 11 sites are shown below and analyzed in the Results section.

Province	Local Site	Geographic Coordinates	Time of Sampling	Population Code
Hunan	Zhoujia, Guiyang County	112.72 E 25.80 N	2019.04.17	GY
Hunan	Xiangyangqiao, Hengnan County	112.73 E 26.72 N	2019.2.23	HN
Hunan	Huamenlou, Xiangxiang City	112.05 E 27.86 N	2019.04.17	XX
Hunan	Baomin Embankment, Yuanjiang City	112.42 E 28.79 N	2019.04.08	YJ
Hunan	Songmuqiao, Huarong County	112.70 E 29.54 N	2019.04.17	HR
Hunan	Langzhou Road, Wuling District	111.70 E 29.09 N	2019.04.18	WL1
Hunan	Nanhupu, Wuling District	111.62 E 29.03 N	2019.04.18	WL2
Hunan	East of District Government, Yongding District	110.54 E 29.12 N	2019.04.10	YD1
Hunan	Houping, Yongding District	110.43 E 29.12 N	2019.04.11	YD2
Hunan	Xinglong Street, Longshan County	109.45 E 29.46 N	2019.04.26	LS
Hubei	Laifeng County Government	109.42 E 29.50 N	2019.04.25	LF

**Table 2:** Information about the seven SSR marker loci, including primer sequences, amplified fragment length variations, allele number, and type of repeat sequence.

Locus	Primer Sequence (5′ -> 3′)	Sizes of SSR Alleles (bp) among the 354 Isolates	Allele Number in Population	Repeated Sequence in Reference Genome
CM15	FAM-ACTGAAGACGGAAGAAGCCA CTTGGCGTTCAAAAGGAGAG	202, 208, 210, 212, 214, 216, 218, 220, 232, 233	10	(AG)12
CM18	HEX-GTGAAGGTCTTTGAGGCGAG AGAAGCCCCCTAAAGCTCAG	139, 142, 145, 149, 152, 153, 155, 158, 161, 164, 167, 170, 173, 176, 179, 182, 185, 191	18	(TCC)10
CM22	FAM-GTTGATCAATTCCCGCTGAT AATGGACCGAAAGATTGACG	233, 239, 241, 243, 245, 246, 249, 251, 253, 256, 257, 260, 263, 264, 267, 268, 271, 272, 273, 276, 282, 283, 289, 290, 296, 300, 304, 316, 319, 323, 331, 335, 354, 357, 364, 398	36	(GAAA)9
CM23	HEX-ACTCTCCAGCGCCGTATCTA TGACCTTAACGATGCAGCAG	216, 217, 220, 228, 232, 236	6	(CTGGG)5
CM25	FAM_CAATGCTTGCTTTCTCCACA CATTGAGATCGAGCAGGACA	128, 140, 145, 146, 152, 157, 162, 168, 173, 183, 188, 193, 198, 200, 209, 215, 225	17	(ATGTT)5
CM27	HEX-GTCGATCACAGACGGGTTCT CTTTGGTTGCGATAAGCCAT	245, 250, 255, 257, 262, 268, 274, 279, 280, 285, 316, 322, 358	13	(ATGAAT)6
CM29	FAM-ATTACATCAGGTGGCGGAAG CCTTGTTTGCACAGCCAGTA	248, 250, 255, 261, 266, 272, 277, 283, 288, 294, 299, 305, 323, 335, 341	15	(TTAGGG)15

**Table 3:** Number of *F. commune* isolates obtained and analyzed from each geographic site and each root tissue type. The detailed root tissue type definitions are provided in Materials and Methods.

Geographic Site	Fibrous Root	Main Root Epidermis	Main Root Pith	Total
GY	20	14	6	40
HN	12	2	6	20
XX	27	24	5	56
YJ	17	11	3	31
HR	15	7	5	27
WL1	33	19	14	66
WL2	11	8	5	24
YD1	10	6	1	17
YD2	7	7	2	16
LS	3	4	4	11
LF	19	18	9	46
Total	174	120	60	354

**Table 4:** Allelic diversity within 11 local populations of *F. commune* causing strawberry root rot in southcentral China.

Population	GY	HN	HR	LF	LS	WL1	WL2	XX	YD1	YD2	YJ
Na	7.857	4.286	4.286	6.429	3.714	7.429	5.143	5.714	3.286	4.286	7.286
Na (Freq. ≥5%)	4.857	4.286	3.143	3.714	3.714	4.286	4.000	3.571	3.286	4.286	4.571
Ne	2.906	2.896	2.456	3.420	2.464	4.101	3.381	2.661	1.821	2.872	4.258
I	1.386	1.086	1.026	1.337	1.002	1.533	1.263	1.138	0.722	1.140	1.585
No. Private Alleles	0.714	0.429	0.286	0.429	0.143	1.286	0.571	0.571	0.286	0.000	1.000
No. LComm Alleles (≤25%)	0.286	0.429	0.143	0.286	0.143	0.571	0.286	0.429	0.429	0.286	0.714
No. LComm Alleles (≤50%)	1.857	1.286	0.714	1.571	1.143	2.000	1.429	2.000	0.714	1.286	2.000
He	0.619	0.566	0.542	0.649	0.548	0.724	0.619	0.560	0.396	0.599	0.732
uHe	0.627	0.580	0.552	0.656	0.574	0.729	0.632	0.565	0.408	0.619	0.744

Na = number of different alleles; Na (Freq. ≥5%) = number of different alleles with a frequency ≥5%; Ne = number of effective alleles = 1/(Sum pi^2^); I = Shannon’s diversity index = −Sum(pi * Ln (pi)); No. Private Alleles = number of alleles unique to a single population; No. LComm Alleles (≤25%) = number of locally common alleles (Freq. ≥5%) found in 25% or fewer populations; No. LComm Alleles (≤50%) = number of locally common alleles (Freq. ≥5%) found in 50% or fewer populations; He = expected heterozygosity = 1 − Sum pi^2^; uHe = unbiased expected heterozygosity = (2N/(2N − 1)) * He. The sign “*” here in the table note represents multiplication.

**Table 5:** AMOVA results for both the total sample and the clone-corrected samples. In the clone-corrected sample, only one strain of each genotype is included in each geographic population for analyses.

Data Type	Sample Category	Source of Variation	df	SS	MS	Est. Var.	%
Original data	Geographic	Among Pops	10	508.374	50.837	1.340	13% ***
		Within Pops	343	3059.166	8.919	8.919	87% ***
		Total	353	3567.540		10.258	100%
	Root tissue	Among Pops	2	33.657	16.829	0.062	1%
		Within Pops	351	3533.882	10.068	10.068	99% ***
		Total	353	3567.540		10.130	100%
Clone-corrected	Geographic	Among Pops	10	208.195	20.819	0.571	6% ***
		Within Pops	205	2009.828	9.804	9.804	94% ***
		Total	215	2218.023		10.375	100%
	Root tissue	Among Pops	2	167.173	8.359	0.000	0%
		Within Pops	213	2050.851	30.779	10.629	100% ***
		Total	215	2218.023		10.629	100%

df: degree of freedom; SS: sum of squares; MS: mean square; Est. Var.: estimated variance; %: percent of total variance. ***, *p* value = 0.001.

**Table 6:** Genetic differentiation (*F_ST_*) between pairs of geographic populations of *F. commune* causing strawberry root rot in southcentral China. The analysis here is based on a total sample of 354 isolates.

Populations	GY	HN	HR	LF	LS	WL1	WL2	XX	YD1	YD2	YJ
GY		0.001	0.001	0.001	0.001	0.001	0.001	0.001	0.001	0.001	0.001
HN	0.135		0.001	0.001	0.001	0.001	0.001	0.001	0.001	0.001	0.001
HR	0.067	0.108		0.001	0.001	0.001	0.001	0.001	0.001	0.001	0.001
LF	0.112	0.137	0.157		0.001	0.001	0.001	0.001	0.001	0.001	0.001
LS	0.115	0.149	0.133	0.089		0.001	0.017	0.001	0.001	0.001	0.001
WL1	0.066	0.098	0.104	0.068	0.078		0.001	0.001	0.001	0.001	0.001
WL2	0.095	0.113	0.148	0.059	0.036	0.071		0.001	0.001	0.001	0.002
XX	0.201	0.157	0.185	0.120	0.141	0.146	0.183		0.001	0.001	0.001
YD1	0.315	0.303	0.310	0.312	0.284	0.227	0.273	0.333		0.001	0.001
YD2	0.058	0.139	0.111	0.090	0.095	0.079	0.098	0.207	0.313		0.001
YJ	0.072	0.087	0.118	0.044	0.075	0.035	0.031	0.143	0.215	0.059	

Note: Values in top right half are *p* values based on 1000 randomizations; values in bottom left half are pairwise *F_ST_* values between pairs of local populations.

**Table 7:** Genetic differentiation (*F_ST_*) between pairs of geographic populations of *F. commune* causing strawberry root rot in southcentral China. The analysis here is based on clone-corrected sample of 215 isolates.

Population	GY	HN	HR	LF	LS	WL1	WL2	XX	YD1	YD2	YJ
GY		0.002	0.020	0.013	0.003	0.032	0.035	0.001	0.001	0.150	0.194
HN	0.057		0.019	0.001	0.002	0.001	0.001	0.005	0.001	0.001	0.001
HR	0.029	0.045		0.001	0.005	0.004	0.001	0.001	0.002	0.080	0.001
LF	0.019	0.081	0.070		0.007	0.004	0.006	0.001	0.001	0.002	0.010
LS	0.053	0.083	0.071	0.047		0.011	0.036	0.002	0.001	0.002	0.005
WL1	0.012	0.050	0.032	0.023	0.036		0.004	0.001	0.001	0.003	0.051
WL2	0.015	0.090	0.072	0.028	0.034	0.029		0.001	0.001	0.001	0.043
XX	0.057	0.035	0.070	0.044	0.057	0.049	0.090		0.001	0.001	0.001
YD1	0.165	0.200	0.099	0.214	0.198	0.120	0.214	0.176		0.001	0.001
YD2	0.010	0.079	0.028	0.046	0.072	0.037	0.079	0.076	0.203		0.009
YJ	0.005	0.076	0.052	0.019	0.053	0.010	0.016	0.047	0.152	0.035	

Note: Values in top right half are *p* values based on 1000 randomizations; values in bottom left half are pairwise *F_ST_* values between pairs of local populations.

## Data Availability

All data in this manuscript, including GenBank sequence accession numbers, are provided in the main manuscript file or in the Supplementary Materials.

## Supplementary Materials

The following supporting information can be downloaded at: https://www.mdpi.com/article/10.3390/genes13050899/s1, Figure S1: Example of SSR allele scoring using GeneMapper. Figure S2: Relationships among 354 *Fusarium commune* strains based on *tef1α* sequences. Figure S3: Relationships among 354 *Fusarium commune* strains based on data at seven SSR loci. Figure S4: Distributions of geographic strains among six genetic clusters (*k* = 6) with each color representing a different genetic cluster. The population labels of 1–11 on the X-axis correspond to GY, HN, HR, LF, LS, WL1, WL2, XX, YD1, YD2, and YJ respectively. Table S1: Information about the 30 tested SSR markers using eight strains of *Fusarium commune*. Table S2: Isolate ID, geographic origin, source site, and SSR genotype of all 354 *Fusarium commune* strains from strawberry root rot samples in southcentral China. Table S3: Distributions of multilocus genotypes of *Fusarium commune* within and between individual local populations.
