# Supplementary material for: Genetic Diversity and Population Structure of Fusarium commune Causing Strawberry Root Rot in Southcentral China
Source: Genes (Basel). 2022 May 18;13(5):899. doi: 10.3390/genes13050899 (PMC9140712; doi:10.3390/genes13050899)
Supplement: Supplementary file 1 [file genes-13-00899-s001.zip › genes-1696969-supplementary/Supplementary_Tables S2 and S3.pdf]

Supplementary Table S2. Distributions of multilocus genotypes of *Fusarium commune* within and between individual local populations.

Isolate ID	GenBank accession number	Original strain ID	Multilocus SSR genotype	Clone correction status	Root tissue type	Population code	Detailed geographic name
G1-A-GY	MZ041792	CZ-1-1	#30	abandon	A	GY	Zhoujia Town, Guiyang County, Chenzhou City
G2-A-GY	MZ041793	CZ-1-12	#6	SAVE	A	GY	Zhoujia Town, Guiyang County, Chenzhou City
G3-A-GY	MZ041794	CZ-1-13	#122	SAVE	A	GY	Zhoujia Town, Guiyang County, Chenzhou City
G4-A-GY	MZ041795	CZ-1-17	#65	SAVE	A	GY	Zhoujia Town, Guiyang County, Chenzhou City
G5-A-GY	MZ041796	CZ-1-18	#3	SAVE	A	GY	Zhoujia Town, Guiyang County, Chenzhou City
G6-A-GY	MZ041797	CZ-1-2	#12	SAVE	A	GY	Zhoujia Town, Guiyang County, Chenzhou City
G7-A-GY	MZ041798	CZ-1-20	#155	SAVE	A	GY	Zhoujia Town, Guiyang County, Chenzhou City
G8-A-GY	MZ041799	CZ-1-23	#103	SAVE	A	GY	Zhoujia Town, Guiyang County, Chenzhou City
G9-A-GY	MZ041800	CZ-1-24	#103	abandon	A	GY	Zhoujia Town, Guiyang County, Chenzhou City
G10-A-GY	MZ041801	CZ-1-25	#103	abandon	A	GY	Zhoujia Town, Guiyang County, Chenzhou City
G11-A-GY	MZ041802	CZ-1-28	#84	SAVE	A	GY	Zhoujia Town, Guiyang County, Chenzhou City
G12-A-GY	MZ041803	CZ-1-36	#112	SAVE	A	GY	Zhoujia Town, Guiyang County, Chenzhou City
G13-A-GY	MZ041804	CZ-1-37	#116	SAVE	A	GY	Zhoujia Town, Guiyang County, Chenzhou City
G14-A-GY	MZ041805	CZ-1-38	#103	abandon	A	GY	Zhoujia Town, Guiyang County, Chenzhou City
G15-A-GY	MZ041806	CZ-1-4	#30	SAVE	A	GY	Zhoujia Town, Guiyang County, Chenzhou City
G16-A-GY	MZ041807	CZ-1-42	#122	abandon	A	GY	Zhoujia Town, Guiyang County, Chenzhou City
G17-A-GY	MZ041808	CZ-1-5	#74	SAVE	A	GY	Zhoujia Town, Guiyang County, Chenzhou City
G18-A-GY	MZ041809	CZ-1-8	#25	SAVE	A	GY	Zhoujia Town, Guiyang County, Chenzhou City
G19-A-GY	MZ041810	CZ-1-9	#6	abandon	A	GY	Zhoujia Town, Guiyang County, Chenzhou City
G20-A-GY	MZ041811	CZ-1-9	#3	abandon	A	GY	Zhoujia Town, Guiyang County, Chenzhou City
G21-B-GY	MZ041812	CZ-2-10	#103	abandon	B	GY	Zhoujia Town, Guiyang County, Chenzhou City

G22-B-GY	MZ041813	CZ-2-12	#103	abandon	B	GY	Zhoujia Town, Guiyang County, Chenzhou City
G23-B-GY	MZ041814	CZ-2-14	#103	abandon	B	GY	Zhoujia Town, Guiyang County, Chenzhou City
G24-B-GY	MZ041815	CZ-2-16	#103	abandon	B	GY	Zhoujia Town, Guiyang County, Chenzhou City
G25-B-GY	MZ041816	CZ-2-17	#2	SAVE	B	GY	Zhoujia Town, Guiyang County, Chenzhou City
G26-B-GY	MZ041817	CZ-2-2	#103	abandon	B	GY	Zhoujia Town, Guiyang County, Chenzhou City
G27-B-GY	MZ041818	CZ-2-25	#124	SAVE	B	GY	Zhoujia Town, Guiyang County, Chenzhou City
G28-B-GY	MZ041819	CZ-2-25	#33	SAVE	B	GY	Zhoujia Town, Guiyang County, Chenzhou City
G29-B-GY	MZ041820	CZ-2-26	#95	SAVE	B	GY	Zhoujia Town, Guiyang County, Chenzhou City
G30-B-GY	MZ041821	CZ-2-27	#96	SAVE	B	GY	Zhoujia Town, Guiyang County, Chenzhou City
G31-B-GY	MZ041822	CZ-2-28	#7	SAVE	B	GY	Zhoujia Town, Guiyang County, Chenzhou City
G32-B-GY	MZ041823	CZ-2-3	#103	abandon	B	GY	Zhoujia Town, Guiyang County, Chenzhou City
G33-B-GY	MZ041824	CZ-2-4	#103	SAVE	B	GY	Zhoujia Town, Guiyang County, Chenzhou City
G34-B-GY	MZ041825	CZ-2-5	#103	abandon	B	GY	Zhoujia Town, Guiyang County, Chenzhou City
G35-C-GY	MZ041826	CZ-3-13	#103	abandon	C	GY	Zhoujia Town, Guiyang County, Chenzhou City
G36-C-GY	MZ041827	CZ-3-14	#65	SAVE	C	GY	Zhoujia Town, Guiyang County, Chenzhou City
G37-C-GY	MZ041828	CZ-3-15	#103	abandon	C	GY	Zhoujia Town, Guiyang County, Chenzhou City
G38-C-GY	MZ041829	CZ-3-16	#156	SAVE	C	GY	Zhoujia Town, Guiyang County, Chenzhou City
G39-C-GY	MZ041830	CZ-3-2	#103	abandon	C	GY	Zhoujia Town, Guiyang County, Chenzhou City
G40-C-GY	MZ041831	CZ-3-3	#103	SAVE	C	GY	Zhoujia Town, Guiyang County, Chenzhou City
G41-A-HN	MZ041832	HY1-1-12	#1	SAVE	A	HN	Xiangyangqiao Town, Hengnan County, Hengyang City
G42-A-HN	MZ041833	HY1-1-13	#8	SAVE	A	HN	Xiangyangqiao Town, Hengnan County, Hengyang City
G43-A-HN	MZ041834	HY1-1-14	#15	SAVE	A	HN	Xiangyangqiao Town, Hengnan County, Hengyang City
G44-A-HN	MZ041835	HY1-1-15	#71	SAVE	A	HN	Xiangyangqiao Town, Hengnan County, Hengyang City
G45-A-HN	MZ041836	HY1-1-16	#44	SAVE	A	HN	Xiangyangqiao Town, Hengnan County, Hengyang City
G46-A-HN	MZ041837	HY1-1-17	#44	abandon	A	HN	Xiangyangqiao Town, Hengnan County, Hengyang City
G47-A-HN	MZ041838	HY1-1-19	#71	abandon	A	HN	Xiangyangqiao Town, Hengnan County, Hengyang City

G48-A-HN	MZ041839	HY1-1-20	#71	abandon	A	HN	Xiangyangqiao Town, Hengnan County, Hengyang City
G49-A-HN	MZ041840	HY1-1-21	#71	abandon	A	HN	Xiangyangqiao Town, Hengnan County, Hengyang City
G50-A-HN	MZ041841	HY1-1-22	#24	SAVE	A	HN	Xiangyangqiao Town, Hengnan County, Hengyang City
G51-A-HN	MZ041842	HY1-1-23	#71	abandon	A	HN	Xiangyangqiao Town, Hengnan County, Hengyang City
G52-A-HN	MZ041843	HY1-1-28	#99	SAVE	A	HN	Xiangyangqiao Town, Hengnan County, Hengyang City
G53-B-HN	MZ041844	HY1-2-12	#99	abandon	B	HN	Xiangyangqiao Town, Hengnan County, Hengyang City
G54-B-HN	MZ041845	HY1-2-14	#99	SAVE	B	HN	Xiangyangqiao Town, Hengnan County, Hengyang City
G55-C-HN	MZ041846	HY1-3-11	#11	SAVE	C	HN	Xiangyangqiao Town, Hengnan County, Hengyang City
G56-C-HN	MZ041847	HY1-3-12	#15	SAVE	C	HN	Xiangyangqiao Town, Hengnan County, Hengyang City
G57-C-HN	MZ041848	HY1-3-13	#99	SAVE	C	HN	Xiangyangqiao Town, Hengnan County, Hengyang City
G58-C-HN	MZ041849	HY1-3-14	#99	abandon	C	HN	Xiangyangqiao Town, Hengnan County, Hengyang City
G59-C-HN	MZ041850	HY1-3-15	#99	abandon	C	HN	Xiangyangqiao Town, Hengnan County, Hengyang City
G60-C-HN	MZ041851	HY1-3-8	#44	SAVE	C	HN	Xiangyangqiao Town, Hengnan County, Hengyang City
G61-A-XX	MZ041852	XT-1-1	#55	SAVE	A	XX	Huamenlou Town, Xiangxiang City, Xiangtan City
G62-A-XX	MZ041853	XT-1-10	#46	SAVE	A	XX	Huamenlou Town, Xiangxiang City, Xiangtan City
G63-A-XX	MZ041854	XT-1-11	#20	SAVE	A	XX	Huamenlou Town, Xiangxiang City, Xiangtan City
G64-A-XX	MZ041855	XT-1-12	#20	abandon	A	XX	Huamenlou Town, Xiangxiang City, Xiangtan City
G65-A-XX	MZ041856	XT-1-13	#20	abandon	A	XX	Huamenlou Town, Xiangxiang City, Xiangtan City
G66-A-XX	MZ041857	XT-1-14	#20	abandon	A	XX	Huamenlou Town, Xiangxiang City, Xiangtan City
G67-A-XX	MZ041858	XT-1-15	#23	SAVE	A	XX	Huamenlou Town, Xiangxiang City, Xiangtan City
G68-A-XX	MZ041859	XT-1-16	#20	abandon	A	XX	Huamenlou Town, Xiangxiang City, Xiangtan City
G69-A-XX	MZ041860	XT-1-17	#20	abandon	A	XX	Huamenlou Town, Xiangxiang City, Xiangtan City
G70-A-XX	MZ041861	XT-1-18	#20	abandon	A	XX	Huamenlou Town, Xiangxiang City, Xiangtan City
G71-A-XX	MZ041862	XT-1-19	#20	abandon	A	XX	Huamenlou Town, Xiangxiang City, Xiangtan City
G72-A-XX	MZ041863	XT-1-2	#20	abandon	A	XX	Huamenlou Town, Xiangxiang City, Xiangtan City
G73-A-XX	MZ041864	XT-1-20	#108	SAVE	A	XX	Huamenlou Town, Xiangxiang City, Xiangtan City

G74-A-XX	MZ041865	XT-1-21	#129	SAVE	A	XX	Huamenlou Town, Xiangxiang City, Xiangtan City
G75-A-XX	MZ041866	XT-1-22	#129	abandon	A	XX	Huamenlou Town, Xiangxiang City, Xiangtan City
G76-A-XX	MZ041867	XT-1-23	#19	SAVE	A	XX	Huamenlou Town, Xiangxiang City, Xiangtan City
G77-A-XX	MZ041868	XT-1-26	#20	abandon	A	XX	Huamenlou Town, Xiangxiang City, Xiangtan City
G78-A-XX	MZ041869	XT-1-27	#56	SAVE	A	XX	Huamenlou Town, Xiangxiang City, Xiangtan City
G79-A-XX	MZ041870	XT-1-3	#18	SAVE	A	XX	Huamenlou Town, Xiangxiang City, Xiangtan City
G80-A-XX	MZ041871	XT-1-33	#14	SAVE	A	XX	Huamenlou Town, Xiangxiang City, Xiangtan City
G81-A-XX	MZ041872	XT-1-4	#21	SAVE	A	XX	Huamenlou Town, Xiangxiang City, Xiangtan City
G82-A-XX	MZ041873	XT-1-4	#51	SAVE	A	XX	Huamenlou Town, Xiangxiang City, Xiangtan City
G83-A-XX	MZ041874	XT-1-5	#21	abandon	A	XX	Huamenlou Town, Xiangxiang City, Xiangtan City
G84-A-XX	MZ041875	XT-1-6	#51	abandon	A	XX	Huamenlou Town, Xiangxiang City, Xiangtan City
G85-A-XX	MZ041876	XT-1-7	#20	abandon	A	XX	Huamenlou Town, Xiangxiang City, Xiangtan City
G86-A-XX	MZ041877	XT-1-8	#20	abandon	A	XX	Huamenlou Town, Xiangxiang City, Xiangtan City
G87-A-XX	MZ041878	XT-1-9	#130	SAVE	A	XX	Huamenlou Town, Xiangxiang City, Xiangtan City
G88-B-XX	MZ041879	XT-2-1	#20	abandon	B	XX	Huamenlou Town, Xiangxiang City, Xiangtan City
G89-B-XX	MZ041880	XT-2-1	#20	abandon	B	XX	Huamenlou Town, Xiangxiang City, Xiangtan City
G90-B-XX	MZ041881	XT-2-10	#20	abandon	B	XX	Huamenlou Town, Xiangxiang City, Xiangtan City
G91-B-XX	MZ041882	XT-2-11	#31	SAVE	B	XX	Huamenlou Town, Xiangxiang City, Xiangtan City
G92-B-XX	MZ041883	XT-2-12	#20	abandon	B	XX	Huamenlou Town, Xiangxiang City, Xiangtan City
G93-B-XX	MZ041884	XT-2-13	#82	SAVE	B	XX	Huamenlou Town, Xiangxiang City, Xiangtan City
G94-B-XX	MZ041885	XT-2-15	#82	abandon	B	XX	Huamenlou Town, Xiangxiang City, Xiangtan City
G95-B-XX	MZ041886	XT-2-16	#82	abandon	B	XX	Huamenlou Town, Xiangxiang City, Xiangtan City
G96-B-XX	MZ041887	XT-2-17	#82	abandon	B	XX	Huamenlou Town, Xiangxiang City, Xiangtan City
G97-B-XX	MZ041888	XT-2-18	#82	abandon	B	XX	Huamenlou Town, Xiangxiang City, Xiangtan City
G98-B-XX	MZ041889	XT-2-19	#20	abandon	B	XX	Huamenlou Town, Xiangxiang City, Xiangtan City
G99-B-XX	MZ041890	XT-2-2	#20	abandon	B	XX	Huamenlou Town, Xiangxiang City, Xiangtan City

G100-B-XX	MZ041891	XT-2-21	#130	abandon	B	XX	Huamenlou Town, Xiangxiang City, Xiangtan City
G101-B-XX	MZ041892	XT-2-22	#47	SAVE	B	XX	Huamenlou Town, Xiangxiang City, Xiangtan City
G102-B-XX	MZ041893	XT-2-24	#39	SAVE	B	XX	Huamenlou Town, Xiangxiang City, Xiangtan City
G103-B-XX	MZ041894	XT-2-26	#139	SAVE	B	XX	Huamenlou Town, Xiangxiang City, Xiangtan City
G104-B-XX	MZ041895	XT-2-27	#39	abandon	B	XX	Huamenlou Town, Xiangxiang City, Xiangtan City
G105-B-XX	MZ041896	XT-2-3	#20	SAVE	B	XX	Huamenlou Town, Xiangxiang City, Xiangtan City
G106-B-XX	MZ041897	XT-2-4	#21	SAVE	B	XX	Huamenlou Town, Xiangxiang City, Xiangtan City
G107-B-XX	MZ041898	XT-2-5	#66	SAVE	B	XX	Huamenlou Town, Xiangxiang City, Xiangtan City
G108-B-XX	MZ041899	XT-2-6	#66	abandon	B	XX	Huamenlou Town, Xiangxiang City, Xiangtan City
G109-B-XX	MZ041900	XT-2-7	#130	SAVE	B	XX	Huamenlou Town, Xiangxiang City, Xiangtan City
G110-B-XX	MZ041901	XT-2-8	#66	abandon	B	XX	Huamenlou Town, Xiangxiang City, Xiangtan City
G111-B-XX	MZ041902	XT-2-9	#22	SAVE	B	XX	Huamenlou Town, Xiangxiang City, Xiangtan City
G112-C-XX	MZ041903	XT-3-14	#29	SAVE	C	XX	Huamenlou Town, Xiangxiang City, Xiangtan City
G113-C-XX	MZ041904	XT-3-16	#82	SAVE	C	XX	Huamenlou Town, Xiangxiang City, Xiangtan City
G114-C-XX	MZ041905	XT-3-18	#82	abandon	C	XX	Huamenlou Town, Xiangxiang City, Xiangtan City
G115-C-XX	MZ041906	XT-3-3	#139	SAVE	C	XX	Huamenlou Town, Xiangxiang City, Xiangtan City
G116-C-XX	MZ041907	XT-3-4	#39	SAVE	C	XX	Huamenlou Town, Xiangxiang City, Xiangtan City
G117-A-YJ	MZ041908	YJ-1-1	#140	SAVE	A	YJ	Baomin Embankment, Yuanjiang City, Yiyang City
G118-A-YJ	MZ041909	YJ-1-13	#49	SAVE	A	YJ	Baomin Embankment, Yuanjiang City, Yiyang City
G119-A-YJ	MZ041910	YJ-1-14	#94	SAVE	A	YJ	Baomin Embankment, Yuanjiang City, Yiyang City
G120-A-YJ	MZ041911	YJ-1-16	#36	SAVE	A	YJ	Baomin Embankment, Yuanjiang City, Yiyang City
G121-A-YJ	MZ041912	YJ-1-17	#134	SAVE	A	YJ	Baomin Embankment, Yuanjiang City, Yiyang City
G122-A-YJ	MZ041913	YJ-1-18	#146	SAVE	A	YJ	Baomin Embankment, Yuanjiang City, Yiyang City
G123-A-YJ	MZ041914	YJ-1-19	#101	SAVE	A	YJ	Baomin Embankment, Yuanjiang City, Yiyang City
G124-A-YJ	MZ041915	YJ-1-20	#101	abandon	A	YJ	Baomin Embankment, Yuanjiang City, Yiyang City
G125-A-YJ	MZ041916	YJ-1-21	#101	abandon	A	YJ	Baomin Embankment, Yuanjiang City, Yiyang City

G126-A-YJ	MZ041917	YJ-1-22	#83	SAVE	A	YJ	Baomin Embankment, Yuanjiang City, Yiyang City
G127-A-YJ	MZ041918	YJ-1-26	#37	SAVE	A	YJ	Baomin Embankment, Yuanjiang City, Yiyang City
G128-A-YJ	MZ041919	YJ-1-28	#5	SAVE	A	YJ	Baomin Embankment, Yuanjiang City, Yiyang City
G129-A-YJ	MZ041920	YJ-1-30	#141	SAVE	A	YJ	Baomin Embankment, Yuanjiang City, Yiyang City
G130-A-YJ	MZ041921	YJ-1-4	#132	SAVE	A	YJ	Baomin Embankment, Yuanjiang City, Yiyang City
G131-A-YJ	MZ042022	YJ-1-6	#147	SAVE	A	YJ	Baomin Embankment, Yuanjiang City, Yiyang City
G132-A-YJ	MZ041922	YJ-1-8	#153	SAVE	A	YJ	Baomin Embankment, Yuanjiang City, Yiyang City
G133-A-YJ	MZ041923	YJ-1-9	#126	SAVE	A	YJ	Baomin Embankment, Yuanjiang City, Yiyang City
G134-B-YJ	MZ041924	YJ-2-1	#58	SAVE	B	YJ	Baomin Embankment, Yuanjiang City, Yiyang City
G135-B-YJ	MZ041925	YJ-2-11	#13	SAVE	B	YJ	Baomin Embankment, Yuanjiang City, Yiyang City
G136-B-YJ	MZ041926	YJ-2-12	#13	abandon	B	YJ	Baomin Embankment, Yuanjiang City, Yiyang City
G137-B-YJ	MZ041927	YJ-2-15	#61	SAVE	B	YJ	Baomin Embankment, Yuanjiang City, Yiyang City
G138-B-YJ	MZ041928	YJ-2-16	#68	SAVE	B	YJ	Baomin Embankment, Yuanjiang City, Yiyang City
G139-B-YJ	MZ041929	YJ-2-17	#153	SAVE	B	YJ	Baomin Embankment, Yuanjiang City, Yiyang City
G140-B-YJ	MZ041930	YJ-2-18	#154	SAVE	B	YJ	Baomin Embankment, Yuanjiang City, Yiyang City
G141-B-YJ	MZ041931	YJ-2-19	#62	SAVE	B	YJ	Baomin Embankment, Yuanjiang City, Yiyang City
G142-B-YJ	MZ041932	YJ-2-25	#63	SAVE	B	YJ	Baomin Embankment, Yuanjiang City, Yiyang City
G143-B-YJ	MZ041933	YJ-2-26	#100	SAVE	B	YJ	Baomin Embankment, Yuanjiang City, Yiyang City
G144-B-YJ	MZ041934	YJ-2-9	#64	SAVE	B	YJ	Baomin Embankment, Yuanjiang City, Yiyang City
G145-C-YJ	MZ041935	YJ-3-11	#83	SAVE	C	YJ	Baomin Embankment, Yuanjiang City, Yiyang City
G146-C-YJ	MZ041936	YJ-3-12	#83	abandon	C	YJ	Baomin Embankment, Yuanjiang City, Yiyang City
G147-C-YJ	MZ041937	YJ-3-9	#36	SAVE	C	YJ	Baomin Embankment, Yuanjiang City, Yiyang City
G148-A-HR	MZ041938	HR-1-17	#10	SAVE	A	HR	Songmuqiao Town, Huarong County, Yueyang City
G149-A-HR	MZ041939	HR-1-17	#104	SAVE	A	HR	Songmuqiao Town, Huarong County, Yueyang City
G150-A-HR	MZ041940	HR-1-20	#104	abandon	A	HR	Songmuqiao Town, Huarong County, Yueyang City
G151-A-HR	MZ041941	HR-1-21	#104	abandon	A	HR	Songmuqiao Town, Huarong County, Yueyang City

G152-A-HR	MZ041942	HR-1-23	#104	abandon	A	HR	Songmuqiao Town, Huarong County, Yueyang City
G153-A-HR	MZ041943	HR-1-24	#104	abandon	A	HR	Songmuqiao Town, Huarong County, Yueyang City
G154-A-HR	MZ041944	HR-1-25	#104	abandon	A	HR	Songmuqiao Town, Huarong County, Yueyang City
G155-A-HR	MZ041945	HR-1-26	#104	abandon	A	HR	Songmuqiao Town, Huarong County, Yueyang City
G156-A-HR	MZ041946	HR-1-27	#72	SAVE	A	HR	Songmuqiao Town, Huarong County, Yueyang City
G157-A-HR	MZ041947	HR-1-28	#104	abandon	A	HR	Songmuqiao Town, Huarong County, Yueyang City
G158-A-HR	MZ041948	HR-1-29	#72	abandon	A	HR	Songmuqiao Town, Huarong County, Yueyang City
G159-A-HR	MZ041949	HR-1-3	#97	SAVE	A	HR	Songmuqiao Town, Huarong County, Yueyang City
G160-A-HR	MZ041950	HR-1-40	#10	abandon	A	HR	Songmuqiao Town, Huarong County, Yueyang City
G161-A-HR	MZ041951	HR-1-52	#44	SAVE	A	HR	Songmuqiao Town, Huarong County, Yueyang City
G162-A-HR	MZ041952	HR-1-6	#109	SAVE	A	HR	Songmuqiao Town, Huarong County, Yueyang City
G163-B-HR	MZ041953	HR-2-12	#10	abandon	B	HR	Songmuqiao Town, Huarong County, Yueyang City
G164-B-HR	MZ041954	HR-2-2	#109	SAVE	B	HR	Songmuqiao Town, Huarong County, Yueyang City
G165-B-HR	MZ041955	HR-2-28	#98	SAVE	B	HR	Songmuqiao Town, Huarong County, Yueyang City
G166-B-HR	MZ041956	HR-2-30	#109	abandon	B	HR	Songmuqiao Town, Huarong County, Yueyang City
G167-B-HR	MZ041957	HR-2-31	#109	abandon	B	HR	Songmuqiao Town, Huarong County, Yueyang City
G168-B-HR	MZ041958	HR-2-32	#109	abandon	B	HR	Songmuqiao Town, Huarong County, Yueyang City
G169-B-HR	MZ041959	HR-2-4	#10	SAVE	B	HR	Songmuqiao Town, Huarong County, Yueyang City
G170-C-HR	MZ041960	HR-3-1	#16	SAVE	C	HR	Songmuqiao Town, Huarong County, Yueyang City
G171-C-HR	MZ041961	HR-3-13	#87	SAVE	C	HR	Songmuqiao Town, Huarong County, Yueyang City
G172-C-HR	MZ041962	HR-3-15	#109	SAVE	C	HR	Songmuqiao Town, Huarong County, Yueyang City
G173-C-HR	MZ041963	HR-3-2	#16	abandon	C	HR	Songmuqiao Town, Huarong County, Yueyang City
G174-C-HR	MZ041964	HR-3-3	#88	SAVE	C	HR	Songmuqiao Town, Huarong County, Yueyang City
G175-A-WL1	MZ042023	CD1-1-1	#75	SAVE	A	WL1	Langzhou Road, Wuling District, Changde City
G176-A-WL1	MZ042024	CD1-1-10	#78	SAVE	A	WL1	Langzhou Road, Wuling District, Changde City
G177-A-WL1	MZ042025	CD1-1-11	#53	SAVE	A	WL1	Langzhou Road, Wuling District, Changde City

G178-A-WL1	MZ042026	CD1-1-12	#53	abandon	A	WL1	Langzhou Road, Wuling District, Changde City
G179-A-WL1	MZ042027	CD1-1-13	#28	SAVE	A	WL1	Langzhou Road, Wuling District, Changde City
G180-A-WL1	MZ042028	CD1-1-14	#26	SAVE	A	WL1	Langzhou Road, Wuling District, Changde City
G181-A-WL1	MZ042029	CD1-1-15	#119	SAVE	A	WL1	Langzhou Road, Wuling District, Changde City
G182-A-WL1	MZ042030	CD1-1-16	#119	abandon	A	WL1	Langzhou Road, Wuling District, Changde City
G183-A-WL1	MZ042031	CD1-1-17	#106	SAVE	A	WL1	Langzhou Road, Wuling District, Changde City
G184-A-WL1	MZ042032	CD1-1-18	#142	SAVE	A	WL1	Langzhou Road, Wuling District, Changde City
G185-A-WL1	MZ042033	CD1-1-19	#26	abandon	A	WL1	Langzhou Road, Wuling District, Changde City
G186-A-WL1	MZ042034	CD1-1-2	#102	SAVE	A	WL1	Langzhou Road, Wuling District, Changde City
G187-A-WL1	MZ042035	CD1-1-20	#53	abandon	A	WL1	Langzhou Road, Wuling District, Changde City
G188-A-WL1	MZ042036	CD1-1-21	#148	SAVE	A	WL1	Langzhou Road, Wuling District, Changde City
G189-A-WL1	MZ042037	CD1-1-22	#32	SAVE	A	WL1	Langzhou Road, Wuling District, Changde City
G190-A-WL1	MZ042038	CD1-1-23	#59	SAVE	A	WL1	Langzhou Road, Wuling District, Changde City
G191-A-WL1	MZ042039	CD1-1-24	#118	SAVE	A	WL1	Langzhou Road, Wuling District, Changde City
G192-A-WL1	MZ042040	CD1-1-25	#40	SAVE	A	WL1	Langzhou Road, Wuling District, Changde City
G193-A-WL1	MZ042041	CD1-1-26	#41	SAVE	A	WL1	Langzhou Road, Wuling District, Changde City
G194-A-WL1	MZ042042	CD1-1-27	#41	abandon	A	WL1	Langzhou Road, Wuling District, Changde City
G195-A-WL1	MZ042043	CD1-1-28	#106	abandon	A	WL1	Langzhou Road, Wuling District, Changde City
G196-A-WL1	MZ042044	CD1-1-29	#123	SAVE	A	WL1	Langzhou Road, Wuling District, Changde City
G197-A-WL1	MZ042045	CD1-1-31	#89	SAVE	A	WL1	Langzhou Road, Wuling District, Changde City
G198-A-WL1	MZ042046	CD1-1-32	#149	SAVE	A	WL1	Langzhou Road, Wuling District, Changde City
G199-A-WL1	MZ042047	CD1-1-34	#79	SAVE	A	WL1	Langzhou Road, Wuling District, Changde City
G200-A-WL1	MZ042048	CD1-1-36	#26	abandon	A	WL1	Langzhou Road, Wuling District, Changde City
G201-A-WL1	MZ042049	CD1-1-37	#32	abandon	A	WL1	Langzhou Road, Wuling District, Changde City
G202-A-WL1	MZ042050	CD1-1-38	#78	abandon	A	WL1	Langzhou Road, Wuling District, Changde City
G203-A-WL1	MZ042051	CD1-1-39	#80	SAVE	A	WL1	Langzhou Road, Wuling District, Changde City

G204-A-WL1	MZ042052	CD1-1-4	#119	abandon	A	WL1	Langzhou Road, Wuling District, Changde City
G205-A-WL1	MZ042053	CD1-1-6	#106	abandon	A	WL1	Langzhou Road, Wuling District, Changde City
G206-A-WL1	MZ042054	CD1-1-8	#106	abandon	A	WL1	Langzhou Road, Wuling District, Changde City
G207-A-WL1	MZ042055	CD1-1-9	#78	abandon	A	WL1	Langzhou Road, Wuling District, Changde City
G208-B-WL1	MZ042056	CD1-2-10	#27	SAVE	B	WL1	Langzhou Road, Wuling District, Changde City
G209-B-WL1	MZ042057	CD1-2-12	#120	SAVE	B	WL1	Langzhou Road, Wuling District, Changde City
G210-B-WL1	MZ042058	CD1-2-13	#27	abandon	B	WL1	Langzhou Road, Wuling District, Changde City
G211-B-WL1	MZ042059	CD1-2-14	#120	abandon	B	WL1	Langzhou Road, Wuling District, Changde City
G212-B-WL1	MZ042060	CD1-2-15	#120	abandon	B	WL1	Langzhou Road, Wuling District, Changde City
G213-B-WL1	MZ042061	CD1-2-16	#106	SAVE	B	WL1	Langzhou Road, Wuling District, Changde City
G214-B-WL1	MZ042062	CD1-2-17	#121	SAVE	B	WL1	Langzhou Road, Wuling District, Changde City
G215-B-WL1	MZ042063	CD1-2-18	#121	abandon	B	WL1	Langzhou Road, Wuling District, Changde City
G216-B-WL1	MZ042064	CD1-2-2	#32	abandon	B	WL1	Langzhou Road, Wuling District, Changde City
G217-B-WL1	MZ042065	CD1-2-20	#107	SAVE	B	WL1	Langzhou Road, Wuling District, Changde City
G218-B-WL1	MZ042066	CD1-2-21	#27	abandon	B	WL1	Langzhou Road, Wuling District, Changde City
G219-B-WL1	MZ042067	CD1-2-22	#90	SAVE	B	WL1	Langzhou Road, Wuling District, Changde City
G220-B-WL1	MZ042068	CD1-2-23	#27	abandon	B	WL1	Langzhou Road, Wuling District, Changde City
G221-B-WL1	MZ042069	CD1-2-24	#120	abandon	B	WL1	Langzhou Road, Wuling District, Changde City
G222-B-WL1	MZ042070	CD1-2-3	#32	SAVE	B	WL1	Langzhou Road, Wuling District, Changde City
G223-B-WL1	MZ042071	CD1-2-6	#45	SAVE	B	WL1	Langzhou Road, Wuling District, Changde City
G224-B-WL1	MZ042072	CD1-2-7	#106	abandon	B	WL1	Langzhou Road, Wuling District, Changde City
G225-B-WL1	MZ042073	CD1-2-8	#120	abandon	B	WL1	Langzhou Road, Wuling District, Changde City
G226-B-WL1	MZ042074	CD1-2-9	#121	abandon	B	WL1	Langzhou Road, Wuling District, Changde City
G227-C-WL1	MZ042075	CD1-3-10	#106	SAVE	C	WL1	Langzhou Road, Wuling District, Changde City
G228-C-WL1	MZ042076	CD1-3-11	#43	SAVE	C	WL1	Langzhou Road, Wuling District, Changde City
G229-C-WL1	MZ042077	CD1-3-13	#121	abandon	C	WL1	Langzhou Road, Wuling District, Changde City

G230-C-WL1	MZ042078	CD1-3-14	#121	abandon	C	WL1	Langzhou Road, Wuling District, Changde City
G231-C-WL1	MZ042079	CD1-3-15	#43	abandon	C	WL1	Langzhou Road, Wuling District, Changde City
G232-C-WL1	MZ042080	CD1-3-17	#106	abandon	C	WL1	Langzhou Road, Wuling District, Changde City
G233-C-WL1	MZ042081	CD1-3-18	#106	abandon	C	WL1	Langzhou Road, Wuling District, Changde City
G234-C-WL1	MZ042082	CD1-3-19	#106	abandon	C	WL1	Langzhou Road, Wuling District, Changde City
G235-C-WL1	MZ042083	CD1-3-2	#121	abandon	C	WL1	Langzhou Road, Wuling District, Changde City
G236-C-WL1	MZ042084	CD1-3-3	#121	SAVE	C	WL1	Langzhou Road, Wuling District, Changde City
G237-C-WL1	MZ042085	CD1-3-6	#143	SAVE	C	WL1	Langzhou Road, Wuling District, Changde City
G238-C-WL1	MZ042086	CD1-3-7	#32	abandon	C	WL1	Langzhou Road, Wuling District, Changde City
G239-C-WL1	MZ042087	CD1-3-8	#32	SAVE	C	WL1	Langzhou Road, Wuling District, Changde City
G240-C-WL1	MZ042088	CD1-3-9	#43	abandon	C	WL1	Langzhou Road, Wuling District, Changde City
G241-A-WL2	MZ042089	CD2-1-10	#115	SAVE	A	WL2	Nanhupu, Wuling District, Changde City
G242-A-WL2	MZ042090	CD2-1-11	#144	SAVE	A	WL2	Nanhupu, Wuling District, Changde City
G243-A-WL2	MZ042091	CD2-1-13	#137	SAVE	A	WL2	Nanhupu, Wuling District, Changde City
G244-A-WL2	MZ042092	CD2-1-14	#135	SAVE	A	WL2	Nanhupu, Wuling District, Changde City
G245-A-WL2	MZ042093	CD2-1-15	#135	abandon	A	WL2	Nanhupu, Wuling District, Changde City
G246-A-WL2	MZ042094	CD2-1-2	#135	abandon	A	WL2	Nanhupu, Wuling District, Changde City
G247-A-WL2	MZ042095	CD2-1-20	#4	SAVE	A	WL2	Nanhupu, Wuling District, Changde City
G248-A-WL2	MZ042096	CD2-1-24	#145	SAVE	A	WL2	Nanhupu, Wuling District, Changde City
G249-A-WL2	MZ042097	CD2-1-28	#135	abandon	A	WL2	Nanhupu, Wuling District, Changde City
G250-A-WL2	MZ042098	CD2-1-3	#99	SAVE	A	WL2	Nanhupu, Wuling District, Changde City
G251-B-WL2	MZ042099	CD2-1-4	#106	SAVE	A	WL2	Nanhupu, Wuling District, Changde City
G252-B-WL2	MZ042100	CD2-2-1	#131	SAVE	B	WL2	Nanhupu, Wuling District, Changde City
G253-B-WL2	MZ042101	CD2-2-10	#145	abandon	B	WL2	Nanhupu, Wuling District, Changde City
G254-B-WL2	MZ042102	CD2-2-11	#145	SAVE	B	WL2	Nanhupu, Wuling District, Changde City
G255-B-WL2	MZ042103	CD2-2-13	#81	SAVE	B	WL2	Nanhupu, Wuling District, Changde City

G256-B-WL2	MZ042104	CD2-2-14	#136	SAVE	B	WL2	Nanhupu, Wuling District, Changde City
G257-B-WL2	MZ042105	CD2-2-6	#148	SAVE	B	WL2	Nanhupu, Wuling District, Changde City
G258-B-WL2	MZ042106	CD2-2-7	#138	SAVE	B	WL2	Nanhupu, Wuling District, Changde City
G259-B-WL2	MZ042107	CD2-2-9	#81	abandon	B	WL2	Nanhupu, Wuling District, Changde City
G260-C-WL2	MZ042108	CD2-3-10	#138	SAVE	C	WL2	Nanhupu, Wuling District, Changde City
G261-C-WL2	MZ042109	CD2-3-11	#136	SAVE	C	WL2	Nanhupu, Wuling District, Changde City
G262-C-WL2	MZ042110	CD2-3-12	#136	abandon	C	WL2	Nanhupu, Wuling District, Changde City
G263-C-WL2	MZ042111	CD2-3-4	#73	SAVE	C	WL2	Nanhupu, Wuling District, Changde City
G264-C-WL2	MZ042112	CD2-3-5	#60	SAVE	C	WL2	Nanhupu, Wuling District, Changde City
G265-A-YD1	MZ042113	ZJJ2-1-10	#52	SAVE	A	YD1	East of District Government, Yongding District, Zhangjiajie City
G266-A-YD1	MZ042114	ZJJ2-1-11	#85	SAVE	A	YD1	East of District Government, Yongding District, Zhangjiajie City
G267-A-YD1	MZ042115	ZJJ2-1-12	#85	abandon	A	YD1	East of District Government, Yongding District, Zhangjiajie City
G268-A-YD1	MZ042116	ZJJ2-1-13	#85	abandon	A	YD1	East of District Government, Yongding District, Zhangjiajie City
G269-A-YD1	MZ042117	ZJJ2-1-13	#85	abandon	A	YD1	East of District Government, Yongding District, Zhangjiajie City
G270-A-YD1	MZ042118	ZJJ2-1-17	#85	abandon	A	YD1	East of District Government, Yongding District, Zhangjiajie City
G271-A-YD1	MZ042119	ZJJ2-1-25	#54	SAVE	A	YD1	East of District Government, Yongding District, Zhangjiajie City
G272-A-YD1	MZ042120	ZJJ2-1-5	#92	SAVE	A	YD1	East of District Government, Yongding District, Zhangjiajie City

G273-A-YD1	MZ042121	ZJJ2-1-7	#85	abandon	A	YD1	East of District Government, Yongding District, Zhangjiajie City
G274-A-YD1	MZ042122	ZJJ2-1-8	#52	abandon	A	YD1	East of District Government, Yongding District, Zhangjiajie City
G275-B-YD1	MZ042123	ZJJ2-2-1	#91	SAVE	B	YD1	East of District Government, Yongding District, Zhangjiajie City
G276-B-YD1	MZ042124	ZJJ2-2-16	#85	abandon	B	YD1	East of District Government, Yongding District, Zhangjiajie City
G277-B-YD1	MZ042125	ZJJ2-2-17	#52	SAVE	B	YD1	East of District Government, Yongding District, Zhangjiajie City
G278-B-YD1	MZ042126	ZJJ2-2-18	#93	SAVE	B	YD1	East of District Government, Yongding District, Zhangjiajie City
G279-B-YD1	MZ042127	ZJJ2-2-19	#85	SAVE	B	YD1	East of District Government, Yongding District, Zhangjiajie City
G280-B-YD1	MZ042128	ZJJ2-2-21	#86	SAVE	B	YD1	East of District Government, Yongding District, Zhangjiajie City
G281-C-YD1	MZ042129	ZJJ2-3-29	#85	SAVE	C	YD1	East of District Government, Yongding District, Zhangjiajie City
G282-A-YD2	MZ042130	ZJJ3-1-1	#57	SAVE	A	YD2	Houping Town, Yongding District, Zhangjiajie City
G283-A-YD2	MZ042131	ZJJ3-1-11	#36	SAVE	A	YD2	Houping Town, Yongding District, Zhangjiajie City
G284-A-YD2	MZ042132	ZJJ3-1-13	#48	SAVE	A	YD2	Houping Town, Yongding District, Zhangjiajie City
G285-A-YD2	MZ042133	ZJJ3-1-2	#17	SAVE	A	YD2	Houping Town, Yongding District, Zhangjiajie City
G286-A-YD2	MZ042134	ZJJ3-1-3	#151	SAVE	A	YD2	Houping Town, Yongding District, Zhangjiajie City
G287-A-YD2	MZ042135	ZJJ3-1-4	#117	SAVE	A	YD2	Houping Town, Yongding District, Zhangjiajie City
G288-A-YD2	MZ042136	ZJJ3-1-5	#117	abandon	A	YD2	Houping Town, Yongding District, Zhangjiajie City
G289-B-YD2	MZ042137	ZJJ3-2-14	#151	SAVE	B	YD2	Houping Town, Yongding District, Zhangjiajie City

G290-B-YD2	MZ042138	ZJJ3-2-14	#151	abandon	B	YD2	Houping Town, Yongding District, Zhangjiajie City
G291-B-YD2	MZ042139	ZJJ3-2-18	#151	abandon	B	YD2	Houping Town, Yongding District, Zhangjiajie City
G292-B-YD2	MZ042140	ZJJ3-2-23	#48	SAVE	B	YD2	Houping Town, Yongding District, Zhangjiajie City
G293-B-YD2	MZ042141	ZJJ3-2-27	#111	SAVE	B	YD2	Houping Town, Yongding District, Zhangjiajie City
G294-B-YD2	MZ042142	ZJJ3-2-28	#113	SAVE	B	YD2	Houping Town, Yongding District, Zhangjiajie City
G295-B-YD2	MZ042143	ZJJ3-2-8	#110	SAVE	B	YD2	Houping Town, Yongding District, Zhangjiajie City
G296-C-YD2	MZ042144	ZJJ3-3-18	#36	SAVE	C	YD2	Houping Town, Yongding District, Zhangjiajie City
G297-C-YD2	MZ042145	ZJJ3-3-3	#157	SAVE	C	YD2	Houping Town, Yongding District, Zhangjiajie City
G298-A-LS	MZ041965	XL-1-24	#9	SAVE	A	LS	Xinglong Street, Longshan County, Xiangxi Autonomous Prefecture
G299-A-LS	MZ041966	XL-1-4	#117	SAVE	A	LS	Xinglong Street, Longshan County, Xiangxi Autonomous Prefecture
G300-A-LS	MZ041967	XL-1-7	#50	SAVE	A	LS	Xinglong Street, Longshan County, Xiangxi Autonomous Prefecture
G301-B-LS	MZ041968	XL-2-11	#70	SAVE	B	LS	Xinglong Street, Longshan County, Xiangxi Autonomous Prefecture
G302-B-LS	MZ041969	XL-2-14	#127	SAVE	B	LS	Xinglong Street, Longshan County, Xiangxi Autonomous Prefecture
G303-B-LS	MZ041970	XL-2-3	#50	SAVE	B	LS	Xinglong Street, Longshan County, Xiangxi Autonomous Prefecture
G304-B-LS	MZ041971	XL-2-7	#67	SAVE	B	LS	Xinglong Street, Longshan County, Xiangxi Autonomous Prefecture
G305-C-LS	MZ041972	XL-3-12	#128	SAVE	C	LS	Xinglong Street, Longshan County, Xiangxi Autonomous Prefecture
G306-C-LS	MZ041973	XL-3-3	#50	abandon	C	LS	Xinglong Street, Longshan County, Xiangxi Autonomous Prefecture

G307-C-LS	MZ041974	XL-3-4	#127	SAVE	C	LS	Xinglong Street, Longshan County, Xiangxi Autonomous Prefecture
G308-C-LS	MZ041975	XL-3-8	#50	SAVE	C	LS	Xinglong Street, Longshan County, Xiangxi Autonomous Prefecture
G309-A-LF	MZ041976	LF-1-10	#131	SAVE	A	LF	Laifeng County Government, Enshi Autonomous Prefecture
G310-A-LF	MZ041977	LF-1-11	#33	SAVE	A	LF	Laifeng County Government, Enshi Autonomous Prefecture
G311-A-LF	MZ041978	LF-1-14	#36	SAVE	A	LF	Laifeng County Government, Enshi Autonomous Prefecture
G312-A-LF	MZ041979	LF-1-16	#150	SAVE	A	LF	Laifeng County Government, Enshi Autonomous Prefecture
G313-A-LF	MZ041980	LF-1-17	#76	SAVE	A	LF	Laifeng County Government, Enshi Autonomous Prefecture
G314-A-LF	MZ041981	LF-1-19	#76	abandon	A	LF	Laifeng County Government, Enshi Autonomous Prefecture
G315-A-LF	MZ041982	LF-1-2	#131	abandon	A	LF	Laifeng County Government, Enshi Autonomous Prefecture
G316-A-LF	MZ041983	LF-1-20	#152	SAVE	A	LF	Laifeng County Government, Enshi Autonomous Prefecture
G317-A-LF	MZ041984	LF-1-24	#38	SAVE	A	LF	Laifeng County Government, Enshi Autonomous Prefecture
G318-A-LF	MZ041985	LF-1-25	#38	abandon	A	LF	Laifeng County Government, Enshi Autonomous Prefecture
G319-A-LF	MZ041986	LF-1-26	#69	SAVE	A	LF	Laifeng County Government, Enshi Autonomous Prefecture
G320-A-LF	MZ041987	LF-1-27	#38	abandon	A	LF	Laifeng County Government, Enshi Autonomous Prefecture
G321-A-LF	MZ041988	LF-1-30	#125	SAVE	A	LF	Laifeng County Government, Enshi Autonomous Prefecture
G322-A-LF	MZ041989	LF-1-31	#42	SAVE	A	LF	Laifeng County Government, Enshi Autonomous Prefecture
G323-A-LF	MZ041990	LF-1-4	#105	SAVE	A	LF	Laifeng County Government, Enshi Autonomous Prefecture
G324-A-LF	MZ041991	LF-1-5	#106	SAVE	A	LF	Laifeng County Government, Enshi Autonomous Prefecture
G325-A-LF	MZ041992	LF-1-6	#105	SAVE	A	LF	Laifeng County Government, Enshi Autonomous Prefecture
G326-A-LF	MZ041993	LF-1-7	#69	abandon	A	LF	Laifeng County Government, Enshi Autonomous Prefecture
G327-A-LF	MZ041994	LF-1-8	#70	SAVE	A	LF	Laifeng County Government, Enshi Autonomous Prefecture
G328-B-LF	MZ041995	LF-2-12	#36	SAVE	B	LF	Laifeng County Government, Enshi Autonomous Prefecture
G329-B-LF	MZ041996	LF-2-13	#33	abandon	B	LF	Laifeng County Government, Enshi Autonomous Prefecture
G330-B-LF	MZ041997	LF-2-14	#34	SAVE	B	LF	Laifeng County Government, Enshi Autonomous Prefecture

G331-B-LF	MZ041998	LF-2-15	#33	abandon	B	LF	Laifeng County Government, Enshi Autonomous Prefecture
G332-B-LF	MZ041999	LF-2-16	#77	abandon	B	LF	Laifeng County Government, Enshi Autonomous Prefecture
G333-B-LF	MZ042000	LF-2-17	#103	SAVE	B	LF	Laifeng County Government, Enshi Autonomous Prefecture
G334-B-LF	MZ042001	LF-2-19	#35	SAVE	B	LF	Laifeng County Government, Enshi Autonomous Prefecture
G335-B-LF	MZ042002	LF-2-2	#114	SAVE	B	LF	Laifeng County Government, Enshi Autonomous Prefecture
G336-B-LF	MZ042003	LF-2-22	#158	SAVE	B	LF	Laifeng County Government, Enshi Autonomous Prefecture
G337-B-LF	MZ042004	LF-2-24	#96	SAVE	B	LF	Laifeng County Government, Enshi Autonomous Prefecture
G338-B-LF	MZ042005	LF-2-3	#126	SAVE	B	LF	Laifeng County Government, Enshi Autonomous Prefecture
G339-B-LF	MZ042006	LF-2-4	#35	abandon	B	LF	Laifeng County Government, Enshi Autonomous Prefecture
G340-B-LF	MZ042007	LF-2-5	#77	SAVE	B	LF	Laifeng County Government, Enshi Autonomous Prefecture
G341-B-LF	MZ042008	LF-2-6	#33	abandon	B	LF	Laifeng County Government, Enshi Autonomous Prefecture
G342-B-LF	MZ042009	LF-2-7	#69	SAVE	B	LF	Laifeng County Government, Enshi Autonomous Prefecture
G343-B-LF	MZ042010	LF-2-8	#35	abandon	B	LF	Laifeng County Government, Enshi Autonomous Prefecture
G344-B-LF	MZ042011	LF-2-8	#33	SAVE	B	LF	Laifeng County Government, Enshi Autonomous Prefecture
G345-B-LF	MZ042012	LF-2-9	#133	SAVE	B	LF	Laifeng County Government, Enshi Autonomous Prefecture
G346-C-LF	MZ042013	LF-3-10	#77	SAVE	C	LF	Laifeng County Government, Enshi Autonomous Prefecture
G347-C-LF	MZ042014	LF-3-11	#76	SAVE	C	LF	Laifeng County Government, Enshi Autonomous Prefecture
G348-C-LF	MZ042015	LF-3-12	#126	SAVE	C	LF	Laifeng County Government, Enshi Autonomous Prefecture
G349-C-LF	MZ042016	LF-3-13	#126	abandon	C	LF	Laifeng County Government, Enshi Autonomous Prefecture
G350-C-LF	MZ042017	LF-3-14	#76	abandon	C	LF	Laifeng County Government, Enshi Autonomous Prefecture
G351-C-LF	MZ042018	LF-3-5	#126	abandon	C	LF	Laifeng County Government, Enshi Autonomous Prefecture
G352-C-LF	MZ042019	LF-3-7	#33	abandon	C	LF	Laifeng County Government, Enshi Autonomous Prefecture
G353-C-LF	MZ042020	LF-3-8	#33	SAVE	C	LF	Laifeng County Government, Enshi Autonomous Prefecture
G354-C-LF	MZ042021	LF-3-9	#126	abandon	C	LF	Laifeng County Government, Enshi Autonomous Prefecture

1, Root tissue type: A: fibrous root; B: main root epidermis; C: main rhizome pith.

Supplementary Tables S3. Distributions of multilocus genotypes of *Fusarium commune* within and between individual local populations.

Geographic population	GY	HN	HR	LF	LS	WL1	WL2	XX	YD1	YD2	YJ
Sample size	40	20	27	46	11	66	24	56	17	16	31
Number of MLGs	20	8	10	22	7	28	15	21	7	10	24
Number of private MLGs	17	6	9	14	5	26	11	21	7	8	22
Number of shared MLGs	3	2	1	8	2	2	4	0	0	2	2
