# Supplementary figures and images for: Genetic Diversity and Population Structure of Fusarium commune Causing Strawberry Root Rot in Southcentral China
Source: Genes (Basel). 2022 May 18;13(5):899. doi: 10.3390/genes13050899 (PMC9140712; doi:10.3390/genes13050899)

## Slide 1
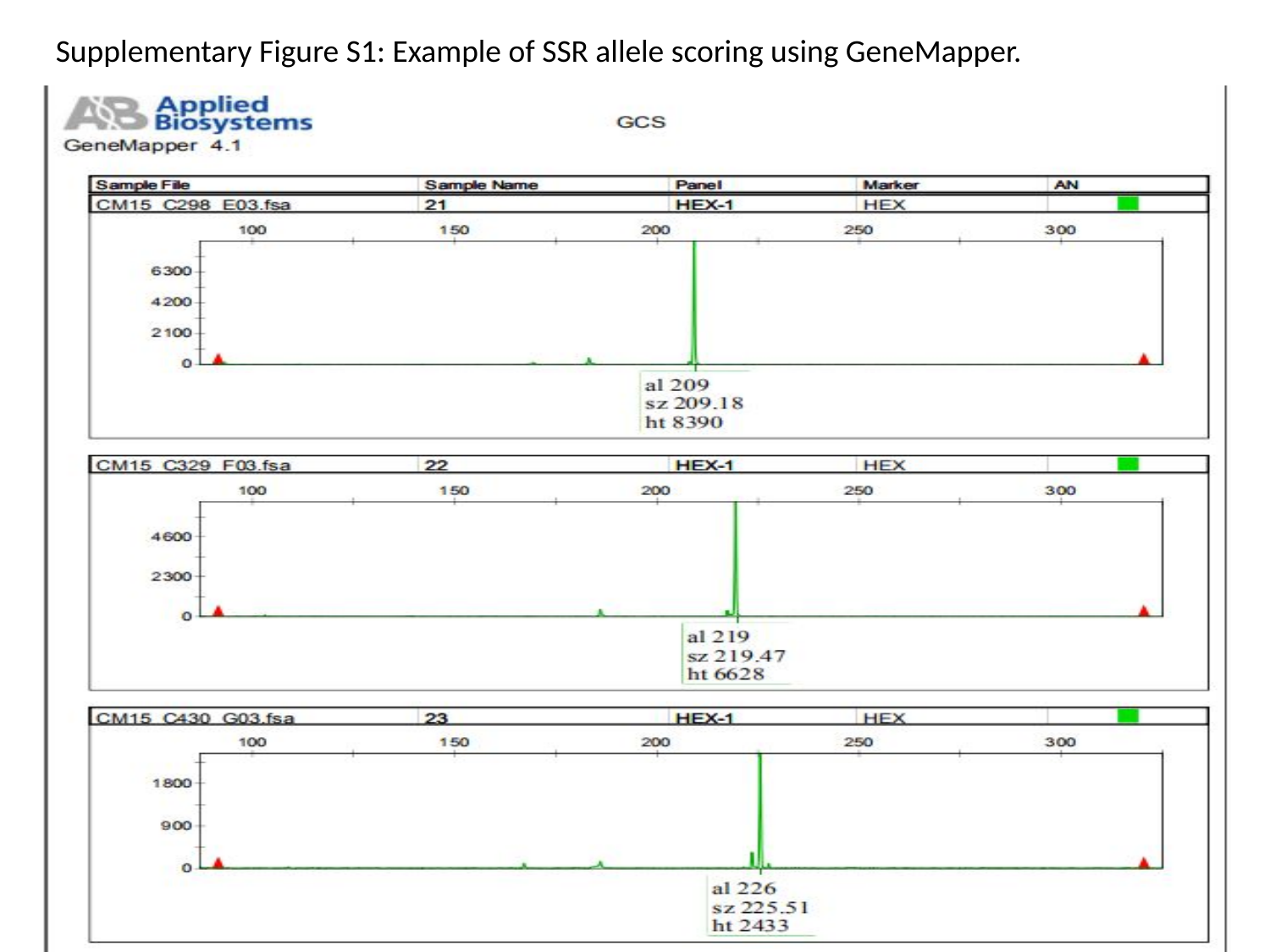

Supplementary Figure S1: Example of SSR allele scoring using GeneMapper.

Supplement: Supplementary file 1 [file genes-13-00899-s001.zip › genes-1696969-supplementary/Supplementary Figure S1.pptx]

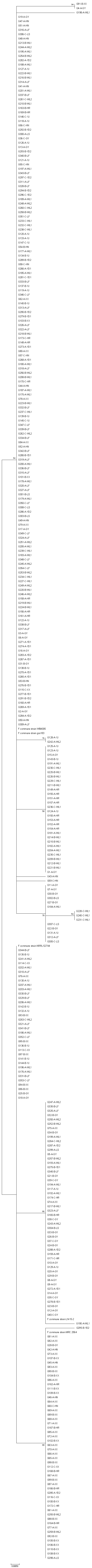

Supplement: Supplementary file 1 [file genes-13-00899-s001.zip › genes-1696969-supplementary/Supplementary Figure S2_tef1_strain relationships.png]

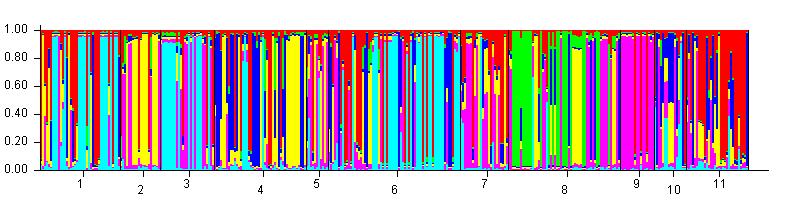

Supplement: Supplementary file 1 [file genes-13-00899-s001.zip › genes-1696969-supplementary/Supplementary Figure S4 (K=6).jpg]
