# Supplementary material for: Genetic Diversity and Population Structure of Fusarium commune Causing Strawberry Root Rot in Southcentral China
Source: Genes (Basel). 2022 May 18;13(5):899. doi: 10.3390/genes13050899 (PMC9140712; doi:10.3390/genes13050899)

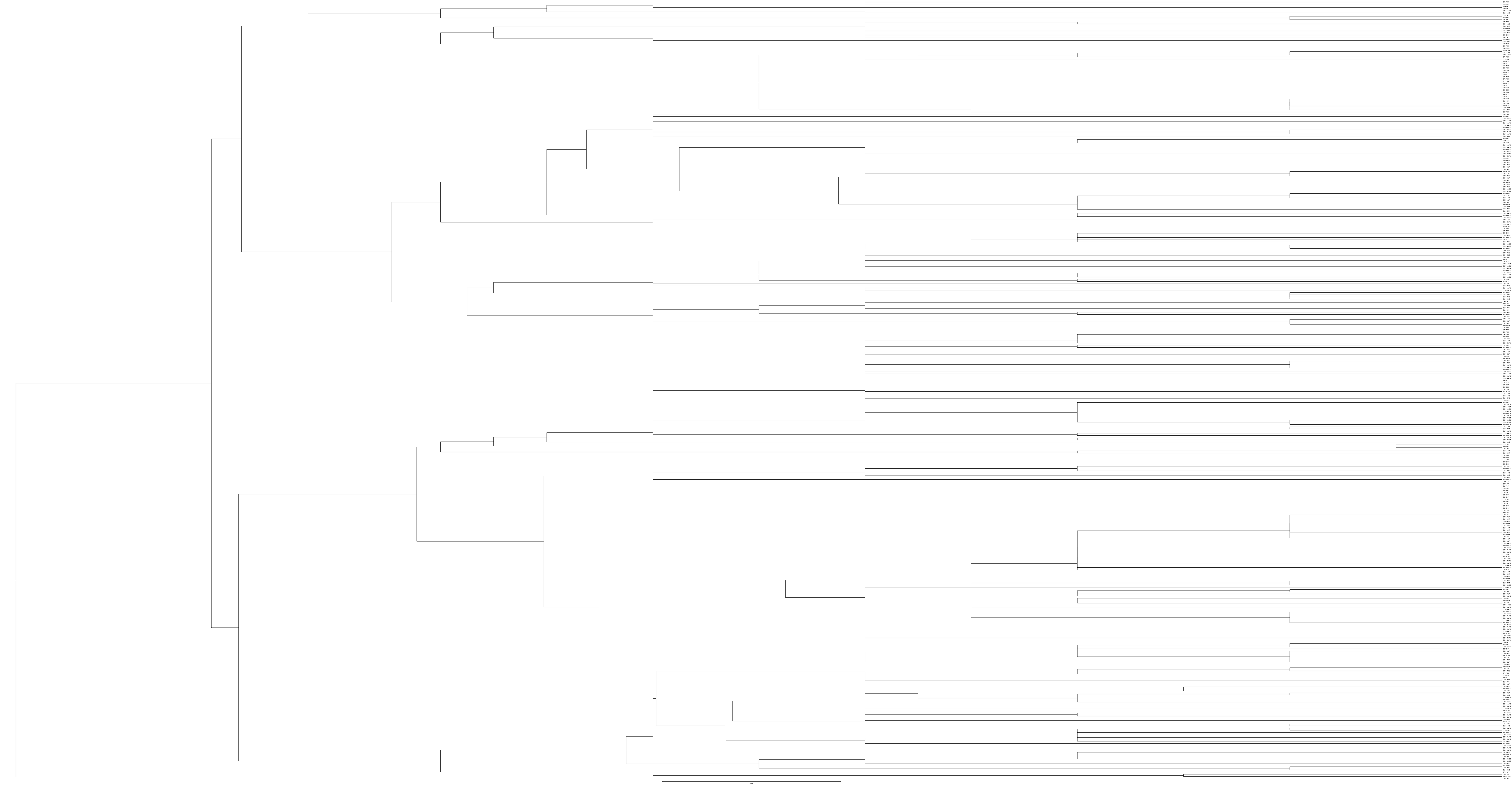

Supplement: Supplementary file 1 [file Supplementary Figure S3_SSR_strain relationships.pdf › genes-1696969-supplementary/Supplementary Figure S3_SSR_strain relationships.pdf]
